# A draft genome of the striped catfish, *Pangasianodon hypophthalmus*, for comparative analysis of genes relevant to development and a resource for aquaculture improvement

**DOI:** 10.1186/s12864-018-5079-x

**Published:** 2018-10-05

**Authors:** Oanh T. P. Kim, Phuong T. Nguyen, Eiichi Shoguchi, Kanako Hisata, Thuy T. B. Vo, Jun Inoue, Chuya Shinzato, Binh T. N. Le, Koki Nishitsuji, Miyuki Kanda, Vu H. Nguyen, Hai V. Nong, Noriyuki Satoh

**Affiliations:** 1 Institute of Genome Research, Vietnam Academy of Science and Technology, Cau Giay, Hanoi, Vietnam; 2 Marine Genomics Unit, Okinawa Institute of Science and Technology Graduate University, Onna, Okinawa, 904-0495 Japan; 3 DNA Sequencing Section, Okinawa Institute of Science and Technology Graduate University, Onna, Okinawa, 904-0495 Japan; 4 Present address: Atmosphere and Ocean Research Institute, The University of Tokyo, Kashiwa, Chiba, 277-8564 Japan

**Keywords:** Striped catfish, Draft nuclear genome, Gadusol biosynthetic genes, Vitamin D-binding protein, cPLA2, Hox cluster, IGF, MHCI, Sex-determination genes, Hypothetical chromosome

## Abstract

**Background:**

The striped catfish, *Pangasianodon hypophthalmus*, is a freshwater and benthopelagic fish common in the Mekong River delta. Catfish constitute a valuable source of dietary protein. Therefore, they are cultured worldwide, and *P. hypophthalmus* is a food staple in the Mekong area. However, genetic information about the culture stock, is unavailable for breeding improvement, although genetics of the channel catfish, *Ictalurus punctatus*, has been reported. To acquire genome sequence data as a useful resource for marker-assisted breeding, we decoded a draft genome of *P. hypophthalmus* and performed comparative analyses.

**Results:**

Using the Illumina platform, we obtained both nuclear and mitochondrial DNA sequences. Molecular phylogeny using the mitochondrial genome confirmed that *P. hypophthalmus* is a member of the family Pangasiidae and is nested within a clade including the families Cranoglanididae and Ictaluridae. The nuclear genome was estimated at approximately 700 Mb, assembled into 568 scaffolds with an N50 of 14.29 Mbp, and was estimated to contain ~ 28,600 protein-coding genes, comparable to those of channel catfish and zebrafish. Interestingly, zebrafish produce gadusol, but genes for biosynthesis of this sunscreen compound have been lost from catfish genomes. The differences in gene contents between these two catfishes were found in genes for vitamin D-binding protein and cytosolic phospholipase A_2_, which have lost only in channel catfish. The Hox cluster in catfish genomes comprised seven paralogous groups, similar to that of zebrafish, and comparative analysis clarified catfish lineage-specific losses of *A5a*, *B10a,* and *A11a*. Genes for insulin-like growth factor (IGF) signaling were conserved between the two catfish genomes. In addition to identification of MHC class I and sex determination-related gene loci, the hypothetical chromosomes by comparison with the channel catfish demonstrated the usefulness of the striped catfish genome as a marker resource.

**Conclusions:**

We developed genomic resources for the striped catfish. Possible conservation of genes for development and marker candidates were confirmed by comparing the assembled genome to that of a model fish, *Danio rerio*, and to channel catfish. Since the catfish genomic constituent resembles that of zebrafish, it is likely that zebrafish data for gene functions is applicable to striped catfish as well.

**Electronic supplementary material:**

The online version of this article (10.1186/s12864-018-5079-x) contains supplementary material, which is available to authorized users.

## Background

Catfish comprise approximately 4000 species belonging to the teleost order Siluriformes [1]. They are globally distributed in fresh, salty, and brackish water. Although catfish have lost their scales evolutionarily, they occupy a phylogenetic position close to cyprinid fishes including the model fish, *Danio rerio* [2, 3]. Catfish are also an Ostariophysian species closely related to zebrafish and carp. Catfish constitute a valuable source of dietary protein [4] and are therefore cultured worldwide as a leading aquaculture species [5–7]. The striped catfish, *Pangasianodon hypophthalmus* Sauvage, 1878, is a freshwater and benthopelagic species that is common and widely cultured in the Mekong River delta [7, 8]. Vietnam is the world’s largest producer of *P. hypophthalmus*, with an estimated 1.1 million tons being cultured on a farming area of more than 5000 ha [9, 10]. However, due to environmental changes and other challenges, aquaculture methods and systems must be constantly examined to improve production. Catfish genomic information may be useful to develop marker-assisted breeding and associated genome-wide analyses for catfish aquaculture.

Genomic information greatly facilitates fundamental research and applications for genetic improvement programs in cultured species [11, 12]. The genomes of several economically important fish species have been sequenced, including Atlantic cod (*Gadus morhua*) [13], rainbow trout (*Oncorhynchus mykiss*) [14], Nile tilapia (*Oreochromis niloticus*) [15], Atlantic salmon (*Salmo salsar*) [16], and channel catfish (*Ictalurus punctatus*) [17]. Using decoded genomes, researchers have analyzed polymorphic markers, linkage maps, and QTL/GWAS (Quantitative Trait Loci/Genome-Wide Association Study). Results of these analyses can be used in breeding programs, including marker-assisted selection (MAS), genome selection (GS), and genome editing. For example, genomic resources for Atlantic salmon have been developed with whole-genome sequences [16] and 9.7 million non-redundant SNPs [18]. Moreover, a high-density genetic linkage map [19] and a number of QTL studies have characterized the correlation between genetic and phenotypic variation, namely, QTLs affecting flesh color and growth-related traits [20–22], late sexual maturation [23], resistance to pancreatic disease (salmonid alphavirus) [24], and resistance to infectious pancreatic necrosis (IPN) [25, 26]. Consequently, MAS has been successfully used in the selection of IPN resistance in Atlantic salmon, which can reduce the number of IPN outbreaks by 75% in salmon farming [27].

Significant efforts have also been devoted to enhancing genomic and genetic research in other economically important aquaculture species, including catfish. The channel catfish, *I. punctatus,* is cultured mostly in the U.S., and its genome has been decoded [11, 17]. The channel catfish genome identified genes relevant to the evolutionary loss of scales in catfish although developmentally relevant genes and genes potentially relevant to aquaculture have not been analyzed in detail. In contrast, less genetic and genomic information has been reported in the striped catfish, *P. hypophthalmus,* which is widely cultured in the Mekong river delta. For example, Sriphairoj et al. [28] were unable to construct sex-specific markers for *Pangasianodon*. Therefore, genomic resources of *P. hypophthalmus* are necessary to develop genome-based technologies for Asian catfish aquaculture. Moreover, *P. hypophthalmus* is naturally distributed in only the Chao Phraya river of Thailand and the Mekong river, which runs through Cambodia, Laos, Thailand, and Vietnam. *P. hypophthalmus* migrates annually between spawning and feeding grounds. This species spawns in the upper reaches of the Cambodian Mekong River, then migrates back to the feeding grounds which are located in the floodplain of Tonle Sap, central and lower Mekong river and the Vietnamese Mekong delta [29]. Genetic diversity of *P. hypophthalmus* remains poorly understood. Only a few studies of population genetics have been done for this species. However, findings are contradictory because of the limited availability of genetic markers [30]. Genomic information about *P. hypophthalmus* is needed for development of molecular markers that can be used in genetic diversity and evolutionary studies.

Here, we report the decoded genome of the striped catfish, *P. hypophthalmus.* We compare the striped catfish genome to the channel catfish and zebrafish reference genomes, because striped catfish are phylogenetically closed to both. We also clarify the conservation of core developmental genes in each lineage. In addition, we try to construct hypothetical chromosomes by anchoring the striped catfish genome to channel catfish chromosomes as a genome sequence resource, although the chromosome number of the striped catfish has been reported as 2n = 60 [31], which is similar to that of channel catfish (2n = 58) [17].

## Results

### Sequencing, assembly, and validation

**Fig. 1:**
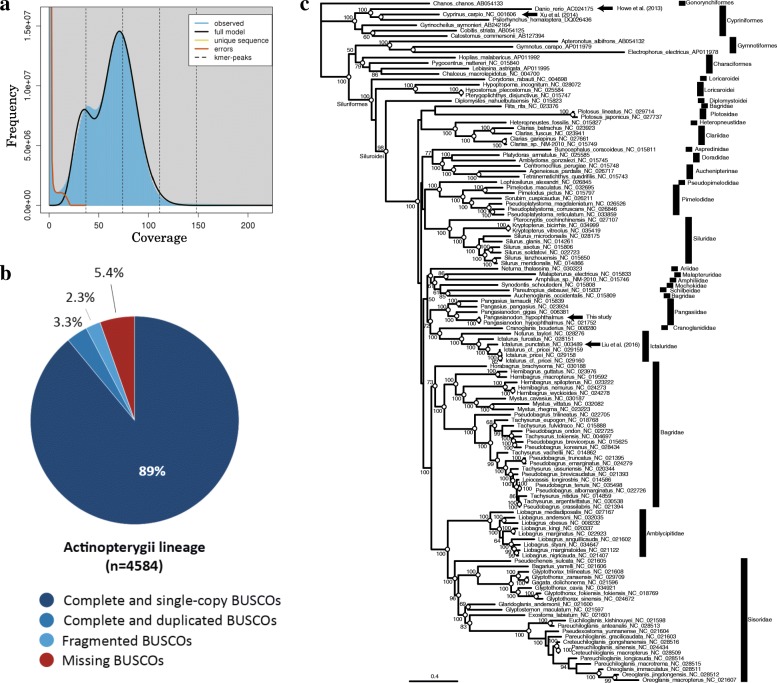
Size estimation of the striped catfish genome and assessment of assembled genome. **a** Paired-end sequences in *P. hypophthalmus* were analyzed using GenomeScope software [78] . The estimated genome size was ~ 700 Mbp, based upon K-mer frequency (K = 41). **b** Assessment of the assembled genome was performed using BUSCO ver. 3. Comparisons with Benchmarking universal single-copy orthologs (BUSCO) sets representing 4584 genes for the Actinopterygii lineage indicated that 92.3% complete BUSCOs were detected in the draft genome, supporting the high quality of genome assembly. **c** Phylogenic position of the sequenced striped catfish was confirmed with an ML tree, which was constructed by comparing 10,665 bp encoding 12 mitochondrial protein genes of 112 species of the order Siluriformes deposited in the NCBI database. The mitochondrial genome sequence of our specimen was almost identical to that of *P. hypophthalmus* decoded in a previous study (NC_021752). The morphological identification of species is confirmed by COX1 gene sequences with voucher numbers (e.g., KU692728 and JF292409 in NCBI). The sister group relationship between two clades (Pangasiidae vs Cranoglanididae/Ictaluridae) was also supported by the previous study [102]. Nodes with white circles were supported by a partition analysis excluding 3rd codon positions (7110 bp). Divergence times between species with decoded genome (arrowheads) were obtained from TIMETREE (http://www.timetree.org): Danio vs Cyprinus (106 Mya), Danio vs Pangasianodon (144 Mya), and Ictalurus vs Pangasianodon (76 Mya)

The genome of a male *Pangasianodon hypophthalmus* was sequenced using Illumina Miseq and Hiseq platforms. The data obtained from two paired-end (PE) and four mate-pair (MP) libraries reached ~ 130 Gb and ~ 350 Gb, respectively (Additional file 1: Table S1). K-mer analysis using PE reads estimated its genome size to be ~ 700 Mbp (Fig. 1a). Data were assembled using a standard pipeline and validated using several software tools (Additional file 2: Figure S1). PE read assembly using Platanus software yielded contigs with an N50 of ~ 6 kbp (Additional file 1: Table S1). Scaffolding with MP reads followed by gap filling resulted in 3304 scaffolds (≥ 1000 bp) with an N50 of 8206 Kbp (Additional file 1: Table S1). The initial assembly was further improved using HaploMerger2. The *P. hypophthalmus* draft genome finally consisted of 568 scaffolds, with an N50 of 14.29 Mbp. This was longer than the scaffold N50 = 7700 kb of the channel catfish genome (estimated size, 1.0 Gbp) [17]. The scaffold total length was ~ 715 Mbp, which corresponded to ~ 102% of the estimated genome.

The GC content of this catfish genome was 38.3%. Repeat masker software showed that interspersed repeats constituted ~ 242 Mbp (~ 33.83% of the draft genome), which was less than that of the zebrafish (52%) [3]. Completeness of genome assembly and annotation was assessed using BUSCO [32]. BUSCO found 89% complete, single-copy orthologs belonging to a ray-finned fish (Actinopterygii) lineage (Fig. 1b). In addition, 90% of RNA-seq data was mapped to the assembled genome (http://catfish.genome.ac.vn, http://marinegenomics.oist.jp/gallery/). Thus, we decoded a high-quality draft genome of *P. hypophthalmus,* which was designated assembly version 2018.

To validate the phylogenetic position of the specimen, we obtained mitochondrial genome sequence data*.* A BLAST search of mitochondrial genes and an analysis of gene order resulted in a single, circular mitochondrial genome that spanned approximately 16.5 kbp and contained 37 genes [33] (Additional file 2: Figure S2). Since the present result was consistent with that of a previous study [34], we used the data for molecular phylogenomics of this fish. We selected 13 protein-coding genes of the mitochondrial genome, and data for the other 112 siluriforms and 14 non-siluriform otocephalans were retrieved from the NCBI database. Using codon-partitioned 10,665 bp data, we estimated a maximum-likelihood (ML) tree according to the analytical procedure shown by Inoue et al. (2010) [35]. We confirmed that our specimen is *P. hypophthalmus* due to the almost identical sequence with that of *P. hypophthalmus* (NC_021752) shown by the short branch lengths between the two species (Fig. 1c). In addition, the clade belonging to *P. hypophthalmus* (Pangasiidae) was grouped with a clade comprising members of the families Cranoglanididae and Ictaluridae. The latter included the channel catfish, *Ictalurus punctatus* [17] (Fig. 1c), which also has a decoded genome, demonstrating that catfishes are closer to cyprinid fishes.

### Genome annotation and assessment of possible lost genes

Using AUGUSTUS software, we predicted protein-coding genes in the draft *P. hypophthalmus* genome. Parameters were determined by training with teleost genes and RNA-seq data of *P. hypophthalmus.* We found 28,580 gene models (gene IDs: phy_g1 to phy_g28580), comparable to genomes of zebrafish and channel catfish (Table 1). The median lengths of genes, exons, and introns were 7316, 119, and 564 bp, respectively (Table 1), which are also comparable to those of other teleosts. The median transcript length was 978 nucleotides, indicating that the striped and channel catfish differ in transcriptome length (Table 1).

**Table 1:** Comparison of the *Pangasianodon hypophthalmus* genome annotation with those of four other fishes

	Actinopterygii				
	Neopterygii				
	Acanthopterygii		Ostariophysi		
	*Oryzias latipes* ^a^	*Takifugu rubripes* ^a^	*Danio rerio* ^a^	*Ictalurus punctatus* ^b^	*Pangasianodon hypophthalmus*
Number of genes	19,686	18,523	26,039	27,395	28,580
Median gene length (bp)	6137	4116	12,342	8668	7316
Median transcripts length (bp)	1242	1311	1741	2769	978
Median exon length (bp)	119	122	124	137	119
Median intron length (bp)	246	142	980	544	564

^a^Data were obtained from Howe et al. [3]

^b^Data were obtained via https://www.ncbi.nlm.nih.gov/genome/annotation_euk/Ictalurus_punctatus/100/

**Fig. 2:**
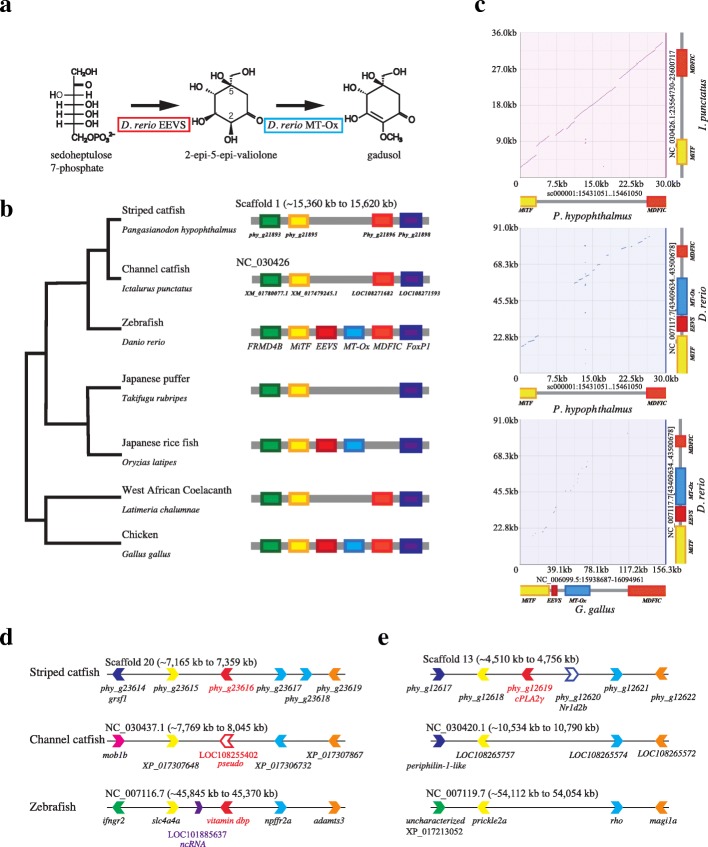
Lineage specific gene losses evaluated from comparisons between the draft genome of the striped catfish and the available genome of the channel catfish, *Icatlurus punctatus*. **a** The catfish lineage lost a gene cluster for sunscreen biosynthesis. The vertebrate sunscreen compound, gadusol, and biosynthetic pathway demonstrated using recombinant zebrafish proteins, EEVS and MT-Ox [39], are shown. **b** Genomic organization of EEVS and MT-Ox-containing region in vertebrates suggests that the catfish *P. hypophthalmus* lost both *EEVS* and *MT-Ox* genes, but arrangements of neighboring genes are conserved. FRMD4B, FERM domain-containing protein 4B. MitF, microphthalmia-associated transcription factor. MDFIC, MyoD-family inhibitor domain-containing protein-like. FoxP1, Forkhead-related transcription factor 1. **c** Comparisons of genomic regions between *MiTF* and *MDFIC* zPicture [103] alignments of *P. hypophathalmus* vs *I. punctatus*, *P. hypophathalmus* vs *D. rerio* and *G. gallus* vs *D. rerio*, respectively. **d** Syntenic regions of catfishes and zebrafish include a pseudogene from a vitamin D-binding protein coding gene in channel catfish. **e** Syntenic regions containing *prikle2a* and *rhodopsin* show the difference between these two catfish genomes. The *cPLA2*γ gene was not found in the channel catfish genome, but was encoded in another region in zebrafish.

Lineage-specific loss of scales has been reported in the channel catfish genome [17]. To evaluate whether the striped catfish genome provides further useful genetic information relative to catfish aquaculture, we surveyed additional gene losses specific to catfish, specifically genes involved in sunscreen biosynthesis. To survive exposure to intense solar radiation, many bacteria, fungi, algae, and marine invertebrates, including corals, produce ultraviolet (UV)-protective compounds, such as mycosporine-like amino acids (MAAs) and related gadusols, [36–38]. Recently, Osborn et al. [39] reported that zebrafish contain the biosynthetic pathway of an ultraviolet-protective compound, gadusol, which is synthesized by two enzymes, EEVS and MT-Ox (Fig. 2a). Genes for the two enzymes are in a tail-to-tail orientation, flanked on the 5′-side by the genes, *FRMD4B* and *MiTF*, and on the 3′-side, by *MDFIC* and *FoxP1* (Fig. 2b). The alignment of the six genes is recognized as a conserved genomic unit in other fish, including Atlantic cod [39]. We identified this synteny in the striped catfish genome, but failed to find the two gadusol-synthetic genes (Fig. 2b). Because the two homologous genes on the 5′-side and the other on the 3’-side were found in the ~ 15.3-Mb- and ~ 15.7-Mb-long scaffold 1, it is likely that both genes were lost in the striped catfish (Fig. 2b). The loss of *MDFIC* in the synteny region of Japanese puffer fish was also evident in this analysis (Fig. 2b). The intergenic region between *MiTF* and *MDFIC* of striped catfish was ~ 20 kbp, which also aligned with the same region of the channel catfish genome (Fig. 2c). These aligned regions show the great similarity between these two catfishes. However, the sequence similarity of those regions between catfish and zebrafish was not confirmed when aligning the intergenic region, as when aligning chick and zebrafish (Fig. 2c). The TblastN search of these intergenic regions using the NCBI database showed partial similarity with reverse transcriptase sequence of zebrafish (BAE46430) and no similarity to *EEVS* and *MT-Ox* genes was found. In addition, no transcriptomes of striped catfish map to the intergenic region. Thus, the genes for EEVS and MT-Ox were most likely lost in the common lineage of two catfishes. Similar gene loss was observed in the west African coelacanth genome [40, 41]. Most catfish are freshwater bottom feeders, and the loss of these genes probably reflects catfish ecology. When catfish are cultured in shallow ponds, limiting UV light exposure may be important for their improved production.

To further assess the usefulness of striped catfish genome, we surveyed 169 genes that were lost from channel catfish, but were found in the armored catfish (the pleco, *Pterygoplichthys pardoralis*, family Loricariidae and the southern striped Raphael, *Platydoras armatulus*, family Doradidae) [17]. Interestingly, differences in two of those genes were detected bewteen striped catfish and channel catfish. These included vitamin D-binding protein coding gene (*dbp*) (Fig. 2d) and cytoplasmic phospholipase A_2_ gene (*cPLA*_*2*_) (Fig. 2e). Vitamin dbp participates in transport of vitamin D metabolites. It is known that cPLA_2_ functions in Golgi membrane tubule function. Thus, striped catfish genome clarified recent lost genes in the channel catfish lineage, indicating its usefulness in comparative genomic analysis.

### Comparative analysis of genes relevant to development

To survey conservation of genes relevant to development, numbers of genes for transcription factors (TF) and signaling molecules (SM) in the *P. hypophthalmus* genome were estimated based on Pfam domain searches (Additional file 1: Tables S2 and S3) and were compared with those of *O. latipes* [42], *T. rubripes* [43], *D. rerio* [3], and *I. punctatus* [17]. TF genes for the SCAN (PF02023) and TBX (PF12598) families were more numerous in the two catfishes than in other fish, suggesting that these gene families have expanded in catfish lineage. Among SM, only the gene family for the MCP signal (PF00015) appeared to have expanded. We confirmed by careful examination that the catfish lineage-specific expansion was not found in the other three fish.

The *Hox* cluster consists of ~ 13 homeodomain-containing transcription factor genes, which show collinearity of expression and function in establishing the antero-posterior body axis and subsequent tissue differentiation [44]. Vertebrates experienced two-rounds of whole genome duplication (2R-WGD) [45–47], although the timing of the first and second rounds is still under debate [48, 49]. Therefore, in contrast to most invertebrates that retain a single Hox cluster, vertebrates contain four paralogous clusters (*HoxA*, *HoxB*, *HoxC*, and *HoxD*) [46, 47]. In addition, teleost fish have experienced one additional round of WGD, known as the teleost-specific WGD (TS-WGD). Therefore, theoretically, teleost genomes have eight paralogous *Hox* clusters (*HoxAa*, *HoxAb*, *HoxBa*, *HoxBb*, *HoxCa*, *HoxCb*, *HoxDa*, and *HoxDb*). However, all teleosts examined to date have seven clusters [50, 51]. The lineage leading to medaka, fugu, and many other fish have lost one of the *HoxC* duplicates, and the lineage represented by zebrafish lost one *HoxD* duplicate. In genome-decoding projects involving metazoans, the presence or absence of *Hox* genes and their clustering have frequently been used to assess proper sequencing and the assembly of their nuclear genomes. Although the *Hox* gene clusters of zebrafish have been analyzed extensively [52], those for catfish have not yet been reported.

**Fig. 3:**
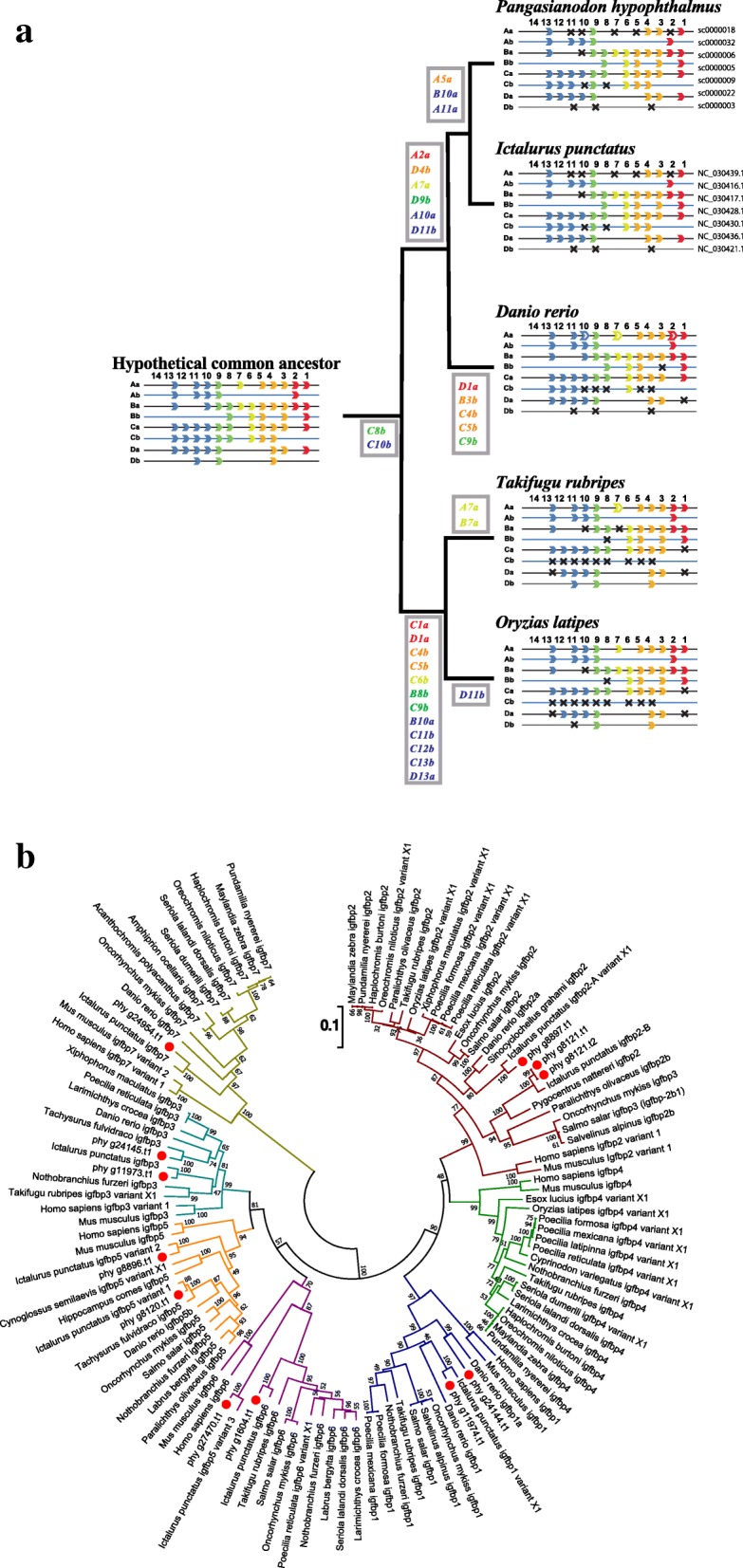
Comparative analysis for development- and growth-related genes. **a** *Hox* clusters from two catfish genomes and a schematic drawing to show possible evolutionary modification of *Hox* cluster genes in the zebrafish/catfish lineage. *Hox* clusters of a hypothetical common ancestor of teleosts (left), *P. hypophthalmus* (upper right), and *Danio rerio* (lower right) are shown. Anterior, middle, and posterior genes are shown in red (1, 2), orange (3 to 5), yellow (6, 7), green (8, 9) and blue (10–13), respectively [78]. Genes in white boxes became pseudogenes, and those lost in the genome are shown with an X. It is likely that a set of *A2a, A7a, A10a, C8b, C10b*, *D4b*, *D9b* and *D11b* were lost in a common ancestor of catfish and *Danio.* In the lineage leading to *Pangasianodon, A5a, A11a* and *B10a* were lost, whereas in the lineage leading to *Danio, B3b, C4b, C5b, C9a,* and *D1a* were lost. *Hox* gene organization for a hypothetical ancestor and *D. rerio* follow the methods of Henkel et al. (2012) [92]. **b** Catfish and zebrafish both retained *IGFP* genes. Molecular phylogenetic analysis of IGFBPs showing conservation and loss of core IGFBPs (1–6). Numbers at nodes indicate bootstrap values

We found that the striped catfish lost one *HoxD* duplicate, similar to zebrafish (Fig. 3a). This suggests that in the context of the seven *Hox* gene cluster, zebrafish and catfish share a common ancestor (Fig. 3a). In relation to the lineage-specific loss of *Hox* genes, Kuraku and Meyer [51] discussed the loss of this *HoxD* duplicate and *HoxA2a, HoxA7a, HoxA10a, HoxC8b, HoxC10b*, *HoxD4b*, *HoxD9b* and *HoxD11b* (Fig. 3a). In the zebrafish, *HoxA2a, HoxA7a,* and *HoxA10a* became pseudogenes, while these genes disappeared in the striped catfish genome. In addition, *HoxB10a* was lost in the striped catfish, but remained intact in the zebrafish. In addition, *HoxC8b* and *HoxC10b* disappeared in the striped catfish, while in the zebrafish, *HoxC4b, HoxC5b* and *HoxC9b* were undetectable. *HoxD1a* was also lost in the zebrafish lineage.

### IGF system

Insulin-like growth factor (IGF) and other molecules associated with this system play pivotal physiological roles in the growth and development of fish, and have been intensively studied [53]. One of the aims of the present study was to identify genes involved in striped catfish growth and link to identify SNPs in these gene correlated with the growth trait in the future to improve catfish aquaculture.

IGF-I and IGF-II are polypeptide hormones of the IGF family. They are structurally homologous to proinsulin, and mature IGF-I and IGF-II exhibit approximately 70% sequence identity. In the *P. hypophthalmus* genome, we identified two genes each for IGF-I and IGF-II (Table 2). These four genes are likely orthologs of *igf-1a*, *−1b*, *−2a*, and *-2b* in zebrafish [54] and located in different scaffolds.

**Table 2:** Genes related to the IGF system in the *Pangasianodon hypophthalmus* genome

Gene family	scaffold number	Gene IDs	Description	nucleotide length	Amino Acid length
IGF	11	phy_g435.t1	insulin-like growth factor I	736	245
IGF	16	phy_g25602.t1	insulin-like growth factor I isoform X1	549	183
IGF	28	phy_g10034.t1	insulin-like growth factor II	639	213
IGF	8	phy_g13975.t1	insulin-like growth factor II	711	237
IGFBP	58	phy_g11974.t1	insulin-like growth factor-binding 1	735	245
IGFBP	42	phy_g24144.t1	insulin-like growth factor-binding 1	796	265
IGFBP	46	phy_g8897.t1	insulin-like growth factor-binding 2A	786	262
IGFBP	3^a^	phy_g8121.t1	insulin-like growth factor-binding 2B	675	225
IGFBP	58	phy_g11973.t1	insulin-like growth factor-binding 3	856	285
IGFBP	42	phy_g24145.t1	insulin-like growth factor-binding 3	906	302
IGFBP	3^a^	phy_g8120.t1	insulin-like growth factor-binding 5	919	306
IGFBP	46	phy_g8896.t1	insulin-like growth factor-binding 5	760	253
IGFBP	9^a^	phy_g1604.t1	insulin-like growth factor-binding 6	612	204
IGFBP	22^a^	phy_g27470.t1	insulin-like growth factor-binding 6	579	193
IGFBP	54	phy_g24954.t1	insulin-like growth factor-binding 7	792	264
IGFR	19	phy_g25100.t1	insulin receptor-like	4026	1342
IGFR	19	phy_g25233.t1	insulin-like growth factor 1 receptor	4057	1352
IGFR	15	phy_g24431.t1	insulin-like growth factor 1 receptor isoform X1	4237	1412

^a^Hox cluster containing scaffolds

IGF-I and IGF-II transmit signals through IGF receptor (IGFR). The IGF-I receptor is a disulfide-linked, heterotetrameric transmembrane protein consisting of two alpha subunits and two beta subunits. Both the α and β subunits are encoded in a single precursor cDNA. In zebrafish, two *igf1r* genes (*igf1ra* and *igf1rb*) are reportedly located on chromosomes 2 and 22, respectively [55]. We found three genes encoding IGFR in the *P. hypophthalmus* genome, all of which are transmembrane proteins (Table 2). Our result suggests one IGFR gene was lost in the zebrafish genome.

IGF-binding proteins (IGFBP) comprise a superfamily that includes six high-affinity IGFBP (core IGFBPs) and at least four additional low-affinity binding proteins, known as IGFBP-related proteins (IGFBP-rP) [56]. Recently, Macqueen et al. [57] identified 20 IGFBP genes of salmonid fish and discussed their evolution in relation to the third and fourth rounds of WGD. We identified 11 IGFBPs in the *P. hypophthalmus* genome, two IGFBP-1s, IGFBP-2a, b, two IGFBP-3s, two IGFBP-5s, two IGFBP-6s, and an IGFBP-7 (Table 2) and examined their molecular phylogenic relationships (Fig. 3b). However, we found no *IGFBP-4* genes in the catfish genomes, which is consistent with the zebrafish genome [58]. This suggests that a common ancestor of catfish and zebrafish lost *IGFBP-4*. Zebrafish retains only nine core IGFBPs, and this lineage likely lost one of its *IGFBP-3*s after it split from the catfish lineage (Fig. 3b).

In the *P. hypophthalmus* genome, two sets of *IGFBP-1* and *IGFBP-3* were tandemly aligned in the same scaffolds. Similarly, two sets of *IGFBP-2* (*IGFBP-2a* or *IGFBP-2b*) and *IGFBP-5* were also tandemly arranged in the same scaffolds. This suggests that *GFBP-1* and *-3*, and *IGFBP-2* and *-5* share an ancestor [59]. Scaffold 3, in which *IGFBP-2b* and *− 5* were located, also contained the *HoxDa* cluster. In addition, two *IGFBP-6s* were closely located to the *HoxCa* and *Cb* clusters, respectively. This provides further support for a previous hypothesis about their relationships [59]. Thus, the striped catfish genome was of sufficient quality to be useful for future syntenic analysis of teleost genomes.

### MHCI genes

Next, we surveyed genes potentially relevant to improvement of aquaculture and breeding. Major histocompatibility complex class I (MHCI) molecules initiate immune responses against invading foreign elements, such as viruses. In teleosts, there are five lineages of MHCI, namely U, Z, S, L and P, which have been classified based on phylogenetic clustering [60]. The number of genes in each lineage differs widely among teleost species. Here, we identified MHCI genes in the *P. hypophthalmus* genome to provide additional data for understanding the complexity of the teleost MHCI and for future studies on genetic variation of genes that may be candidates for development of molecular markers related to disease resistance.

**Fig. 4:**
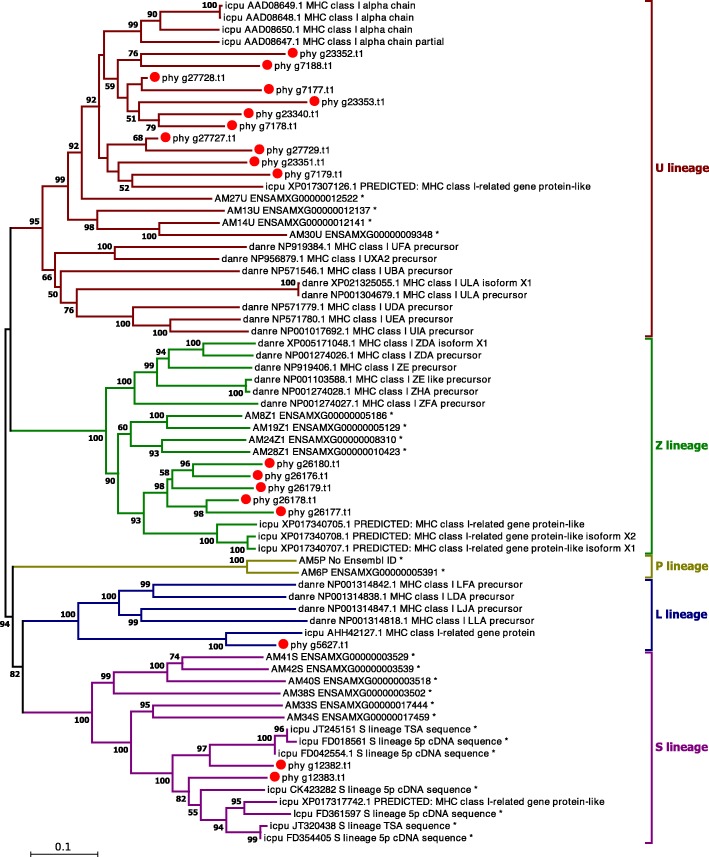
Molecular phylogenetic tree of MHC class I genes. Genes in the U, Z, and S lineages are expanded in the two catfishes, respectively. Numbers at nodes indicate bootstrap values. (Abbreviations: AM = *Astyanyx mexicanus*; danre = *Danio rerio*; Icpu = *Ictalurus punctatus*; phy = *Pangasianodon hypophthalmus*). *MHC class I sequences were used in previous study [60]

In the *P. hypophthalmus* genome, 19 MHCI genes were identified by BLAST searches (Table 3). Of these sequences, 11 genes belong to the U lineage, 5 genes belong to the Z lineage, 2 genes belong to the S lineage, and 1 gene belongs to the L lineage (Fig. 4). This distribution is compatible with what has been reported in previous studies of teleost MHC class I, with genes in the U and Z lineages being more numerous than those in other lineages [60]. The P lineage has not been found in the *P. hypophthalmus* genome.

**Table 3:** The number of MHC Class I lineage genes predicted in the *Pangasianodon hypophthalmus* genome

Predicted MHC Class I lineage^a^	scaffold number	Gene IDs	CDS length
U	sc0000028	phy_g23340.t1	979
U	sc0000028	phy_g23351.t1	2826
U	sc0000028	phy_g23352.t1	1500
U	sc0000028	phy_g23353.t1	2190
U	sc0000013	phy_g27727.t1	355
U	sc0000013	phy_g27728.t1	556
U	sc0000013	phy_g27729.t1	630
U	sc0000006	phy_g7177.t1	1017
U	sc0000006	phy_g7178.t1	1069
U	sc0000006	phy_g7179.t1	534
U	sc0000006	phy_g7188.t1	1807
Z	sc0000105	phy_g26176.t1	1021
Z	sc0000105	phy_g26177.t1	2161
Z	sc0000105	phy_g26178.t1	826
Z	sc0000105	phy_g26179.t1	1180
Z	sc0000307	phy_g26180.t1	1282
S	sc0000004	phy_g12382.t1	946
S	sc0000004	phy_g12383.t1	562
L	sc0000040	phy_g5627.t1	813

^a^MHC Class I lineages are classified according to Grimholt et al. [60]

### Genes related to sex determination

In teleosts, sex determination mechanisms are extremely diverse, differing among closely related species and even within species [61]. Two sex-determining systems, the XY system (i.e., male-heterogamety) and the ZW system (i.e., female-heterogamety), have been found in fish. For example, the XY sex determination system occurs in medaka (*Oryzias latipes*) [62], zebrafish (*D. rerio*) [63] and rainbow trout (*Oncorhynchus mykiss*) [64], while the ZW sex determination system is found in turbot (*Scophthalmus maximus*) [65] and California Yellowtail (*Seriola dorsalis*) [66]. However, sex determination mechanisms in most fish remain unknown. They have been clarified in only a few fish spices. In medaka, a duplicated *Dmrt* gene on the Y-chromosome was found to be a sex determination gene [67]. In rainbow trout, a Y-linked gene (*sdY*) was identified as a sex control gene [64]. In fugu, sex determination is controlled by an SNP in the anti-Mullerian hormone receptor type II (*Amhr2*) gene [68]. In zebrafish, four sex-associated regions (*sar3*, *sar4*, *sar 5* and s*ar16*) have been identified and chromosome 4 is believed to be a sex-chromosome [68]. In aquaculture, sex ratio control is very important because in many economically important fish species, monosex cultures are developed to increase aquaculture production [69]. Genetic information regarding sex determination will enable us to develop sex-linked markers.

In this study, we screened candidate sex-determination genes in the *P. hypophthalmus* genome. BLAST results showed that 15 candidate genes, which were previously reported in zebrafish and channel catfish, were identified in *P. hypophthalmus* (Table 4). However, one of these, the *hsd17b3* gene received low coverage (47%). Channel catfish have the XY system. By analysis of the testis transcriptome, a number of genes, such as *Dmrt1*, *Dmrt2*, *Dmrt3*, *TDRDs*, *PIWIs*, *DDXs,* and *Sox9* were found to be male-biased genes [70]. In a recent study, *Sox30* was also found to be significantly up-regulated in males [71]. Male-biased genes may be involved in sex determination in channel catfish, and channel catfish are supposed to have a polygenic sex determination system, similar to that in zebrafish. In the *P. hypophthalmus* testis transcriptome, transcripts of *Dmrt2*, *Dmrt3*, *hsd17b3*, *and sf1* were not found, while transcripts of *Sox9*, *Sox30*, *TDRD1*, and *spata17* were found with low FPKM (Fragments Per Kilobase Million). Therefore, the number of male-biased genes in the striped catfish may differ from that of channel catfish. Our data provide basic information for further studies of sex-determination genes.

**Table 4:** Candidate genes for sex determination in catfish genomes

	Striped catfish (*Pangasianodon hypophthalmus*)		Channel catfish (*Ictalurus punctatus*)		Zebrafish (*Danio rerio*)	
Gene name	Scaffold number	Gene IDs	Chr	NCBI ID	Chr	Ensemble ID
sox9b	sc0000006	phy_g7559	2	XP_017315164.1	3	ENSDARG00000043923
sox30	sc0000034	phy_g15976	4	XP_017347423.1	6	ENSDARG00000031664
dmrt1	sc0000020	phy_g50867	22	XP_017308041.1	5	ENSDARG00000007349
dmrt2	sc0000020	phy_g50865	22	XP_017308058.1	5	ENSDARG00000015072
dmrt3	sc0000020	phy_g50866	22	XP_017308040.1	5	ENSDARG00000035290
hsd17b3^a^	sc0000001	phy_g48256^a^	5	XP_017323206.1	8	ENSDARG00000023287
sf1	sc0000027	phy_g43702	7	XP_017327018.1	7	ENSDARG00000008188
amh	sc0000055	phy_g41360	10	XP_017333187.1	22	ENSDARG00000014357
tdrd1	sc0000005	phy_g9344	13	XP_017338221.1	12	ENSDARG00000007465
tdrd7	sc0000027	phy_g43892	7	XP_017327887.1	1	ENSDARG00000032808
piwil1	sc0000047	phy_g14817	20	XP_017351061.1	8	ENSDARG00000041699
piwil2	sc0000020	phy_g50577	22	XP_017306734.1	5	ENSDARG00000062601
ddx4	sc0000035	phy_g5089	16	XP_017345559.1	10	ENSDARG00000014373
spata22	sc0000010	phy_g34371	28	XP_017316192.1	5	ENSDARG00000098537
spata17	sc0000014	phy_g35239	9	XP_017330886.1	17	ENSDARG00000054414

^a^BLAST results of the candidate genes for sex determination with at least 50% coverage of the *P. hypophthalmus* gene, except phy_g48256 (47% coverage)

### Construction of hypothetical chromosomes

**Fig. 5:**
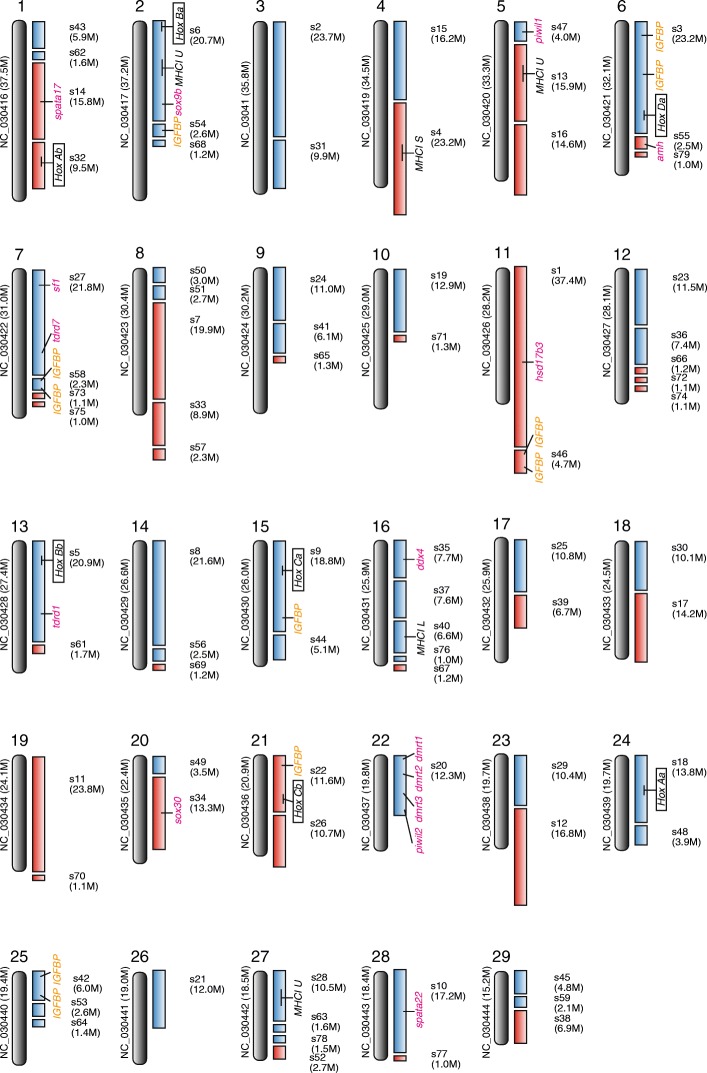
Constructing hypothetical chromosomes of the striped catfish, *P. hypophthalmus.* All scaffolds (> 1 Mb) are anchored to channel catfish chromosomes (*n* = 29). Blue scaffolds show high similarities to one of the channel catfish chromosomes (round gray rectangles), suggesting an orthologous relationship from a common ancestral chromosome. Red scaffolds anchored to the best-hit chromosome imply less conservation from an ancestral chromosome since more than 50% of genes have hits on other channel catfish chromosomes. Some of their scaffolds might be related to differences in the chromosome number (*n* = 30 in *P. hypophthalmus*). Genes related to development and candidate genes for markers are mapped onto striped catfish scaffolds

To make the striped catfish genome a more useful resource, we tried to construct hypothetical chromosomes, based on a comparison with 29 chromosomes of the channel catfish. By our criteria, 58% (417 Mb) of the striped catfish scaffolds mapped to a counterpart on a chromosome of channel catfish (Fig. 5; Additional file 1; Table S6). For example, our analysis indicated scaffold 6 and scaffold 54, which contain *HoxBa* and *IGFBP*, respectively, might be mapped on the same chromosome of striped catfish. Thus, our analysis provides potential linkage groups of the draft genome. Also, scaffold 20, which contains the four sex-determination-related genes (*PIWI12*, *Dmrt1*, *Dmrt2*, and *Dmrt3*), has experienced less inter-chromosomal rearrangement in the catfish lineage (Fig. 5; Table 4). On the other hand, 42% (298 Mb) of striped catfish scaffolds may correspond to genomic regions with higher interchromosomal rearrangement after splitting from common ancestor of the two catfishes (Fig. 4; Additional file 1; Table S6). Thus, this hypothesized genome map of striped catfish will be an important resource for the construction of a physical map in the future.

## Discussion

Comparative analysis of genes that are relevant to development indicated that (1) the draft genome of *P. hypophthalmus* is of comparable quality to other fish genomes, (2) the *Hox* cluster of the catfish is more comparable to that of zebrafish than to those of medaka and other fish, and (3) catfish and zebrafish have experienced common and lineage-specific losses of Hox genes, although the effect is larger in zebrafish than in catfish. Comparison of the *Hox* cluster suggested that the phylogenetic position of striped catfish is closer to zebrafish than to other model fish. Therefore, the *Hox* cluster of *P. hypophthalmus* provides evidence for further discussion of the evolutionary modification of fish *Hox* clusters and TS-WGD. For example, the catfish lineage lost two posterior hox genes after splitting from the zebrafish lineage. This might be related to the special morphology of catfish.

The construction of our hypothetical chromosomes suggested that catfish genomes have experienced more frequent inter-chromosomal rearrangements (Blue scaffolds in Fig. 5) than have invertebrate genomes [72]. The chromosome numbers of channel and striped catfishes are *n* = 29 and *n* = 30, respectively [17, 31]. Therefore, if inter-chromosomal rearrangement is rare, many scaffolds of striped catfish should be anchored on one chromosome of channel catfish. Nonetheless, our comparative genomic analysis of the two catfishes suggests that catfish chromosomes have few inter-chromosomal rearrangement regions (Fig. 5), implying that the channel catfish genome is useful in constructing a physical map of the striped catfish genome. Although sex chromosomes and the sex-determination mechanisms of the catfish are unknown, our hypothetical chromosomes from a male will be useful for analyzing these genomic regions. In a future study, we will identify single nucleotide polymorphisms and polymorphic microsatellites using the striped catfish genome as a reference, and we will prepare a fine linkage or physical map of these data.

## Conclusion

In this study, we developed a genome sequence resource for the striped catfish, *Pangasianodon hypophthalmus*. Possible conservation of genes for transcription factors and signaling molecules was confirmed by comparing the assembled genome to a model fish, *Danio rerio*. Seven *Hox* cluster regions in the catfish and zebrafish genomes contained 51 and 49 genes, respectively, suggesting the conservation of core developmental mechanisms. The striped catfish retained more IGF signaling genes than zebrafish, but the biosynthetic genes for vertebrate sunscreen molecules have been found in the zebrafish genome but not the catfish genome, documenting enzymatic gene loss in this catfish. Altogether, the present whole genome sequence of the *P. hypophthalmus* might be useful as a reference to find SNPs with marker-assisted breeding and associated genome-wide analysis for further aquaculture development of the striped catfish.

## Methods

### Sampling

This study was carried out using striped catfish (*P. hypophthalmus*) from Research Institute of Aquaculture No.2, Vietnam. Genomic DNA was isolated from the testis of an adult male striped catfish. For RNA-seq analyses, fertilized eggs, embryos, and larvae at various developmental stages were collected. Various organs and tissues were also isolated from both female and male adult fishes for RNA-seq analyses. To dissect the tissues, several incisions were made along ventral side and lateral line of the specimen. The fresh tissues were submerged into the RNAlater solution. Details of sampling for transcriptomic analyses are in the NCBI database (the accession nos., SRX3887330-SRX3887334).

### DNA extraction and purification

The testis was powdered in liquid nitrogen and homogenized in DNA extraction buffer (10 mM Tris HCl, pH 8.0; 150 mM EDTA; 1% SDS; 200 μg/mL Proteinase K). DNA was extracted using a phenol-chloroform extraction protocol and pelleted with 100% ethanol. DNA quality and quantity were evaluated by electrophoresis on a 1% agarose gel, and using a NanoDrop spectrophotometer and an Agilent 2100 Bioanalyzer with an Agilent High-Sensitivity DNA Kit.

### DNA library construction and Illumina sequencing

Pair-end (PE) libraries were constructed using a TruSeq DNA PCR-Free Kit (Illumina) according to manufacturer protocols. Mate-pair libraries of 3-kb, 7-kb, 10-kb, and 15-kb fragments were prepared using a Nextera Mate-Pair (MP) Library Preparation Kit (Illumina) following the manufacturer procedure. All pair-end and mate-pair libraries were sequenced using Illumina Miseq and Hiseq 2500 sequencing platforms (Additional file 1: Table S1) with Illumina protocols for whole-genome shotgun sequencing (WGS). PE read length from Miseq was ~ 2 × 310 bp. PE and MP reads from Hiseq 2500 were ~ 2 × 145 bp and ~ 2 × 295 bp, respectively (Additional file 1: Table S1).

### Sequence data processing and genome assembly

Quality of raw sequencing reads was assessed using FastQC v.0.11.5 [73]. Adapter sequences and low-quality reads were trimmed using Trimmomatic v.0.35 [74], PRINSEQ v.0.20.4 [75] and NextClip v1.3 [76], and k-mer analysis was performed using Jellyfish [77]. GenomeScope [78] was applied to estimate genome size. Miseq and Hiseq paired-end reads were assembled de novo with Platanus [79]. Using Illumina mate-pair information, subsequent scaffolding was also performed with Platanus. Gaps in scaffolds were closed using Illumina paired-end data and Platanus software. Completeness of the assembly was estimated with CEGMA v2.5 [80] and Benchmarking Universal Single-Copy Orthologs (BUSCO) v3 [32]. For the post-assembly stage, HaploMerger2 [81] was used to improve the continuity of the initial assembly generated by Platanus. The workflow of the assembly and gene prediction is shown in Fig. S1 (Additional file 2).

### Gene modeling

Simple repeat sequences were identified with RepeatScout v. 1.0.5 [82] and RepeatModeler [83] and masked with RepeatMasker [84]. Masked genome sequences were subjected to produce a gene model or prediction (*Pangasianodon hypophthalmus* Gene Model ver. 2018) with Augustus software [85] and BRAKER2 pipeline [86] with ab initio, homology-based, and EST-based approaches (Additional file 2: Figure S1). For the homology-based approach, protein sequences predicted for *Danio rerio* were aligned using Exonerate v.2.2 [87]. With TopHat2 [88], high-quality RNA-seq reads of *P. hypophthalmus* were used to generate intron hints for EST-based prediction. Details of RNA-seq data are described elsewhere (Oanh T. P. Kim et al., in preparation).

### Genome browser

A genome browser has been established for the assembled sequences using the JavaScript-based genome browser, JBrowse [89]. Its URL is http://marinegenomics.oist.jp/genomes/gallery or http://catfish.genome.ac.vn

### Annotation and identification of genes

Protein-coding genes in the *P. hypophthalmus* genome were surveyed as follows. (i) Nucleotide and amino acid sequences of well-annotated genes of model organisms were used as queries for BLAST searches, including TBLASTN [90] of the *P. hypophthalmus* genome. (ii) Pfam domain searches were performed to identify protein domains included in the putative proteins from all gene models [91] (Pfam-A.hmm, release 24.0).

Hox gene clusters were surveyed based on previous reports of teleost Hox clusters [92, 93]. *Hox* cluster-containing scaffolds from Blast analyses using teleost *Hox* sequences were visualized using a genome browser of *P. hypophthalmus*. Gene model IDs (ver. 2017 and ver. 2018) and transcriptome contigs for *Hox* genes were assigned and confirmed manually (Additional file 1: Table S4).

Genes for the IGF system were screened using a BLAST search and annotated with the BLAST2GO pipeline [94]. For the IGFBP family, the complete salmonid *IGFBP* gene system [57] was also used as a query for BLAST searches of *IGFBP* genes in the *P. hypophthalmus* genome.

MHCI genes in the striped catfish genome were identified based on previous reports [60, 95] and using BLAST searches. Newly identified MHCI genes were aligned with previously reported MHCI genes from different species using the MUSCLE [96] and then based on phylogenetic clustering, MHCI genes were classified into various lineages.

Sex-related genes from zebrafish [63] and channel catfish [70, 71] were used to survey sex-related genes in the striped catfish genome. Based on BLAST searches, candidate sex determination genes and gene-containing scaffolds were identified.

### Molecular phylogeny

With BLAST searches, mitochondrial genome sequences in the draft genome (ver. 2018) of *P. hypophthalmus* were surveyed using mitochondrial genes (NC-021752) as a query. The resultant sequence was confirmed with NOVOplasty [97]. Maximum-likelihood (ML) analysis using RAxML v. 7.2.4 [98] was performed and a tree was constructed as previously described [35].

Newly identified *IGFBP* genes from *P. hypophthalmus* and *IGFBP* genes from different taxa available in the NCBI Nucleotide database (Additional file 1: Table S5) were used for phylogenetic analysis. Multiple alignment of *IGFBP* sequences was performed using the MAFFT web-based tool [99] with default parameters. A phylogenetic tree for IGFBPs was constructed with MEGA7.0 [100] using neighbor-joining methods [101]. The tree topology was evaluated with a bootstrap probability calculated on 1000 resamplings. We applied the same method for phylogenetic tree construction of MHCI genes.

### Anchoring the striped catfish scaffolds to channel catfish chromosomes

To anchor scaffolds on chromosomes of the channel catfish, 28,580 gene models of the striped catfish are used as queries by BLASTN. If a scaffold had better than 50% gene matches on a chromosome, it was hypothesized to have come from a common ancestral chromosome between channel catfish and striped catfish. If a scaffold had less than 50% hit on a chromosome, the scaffold was classified as a less conserved region.

## Additional files

Additional file 1: **Table S1.** Summary of Miseq and Hiseq reads of striped catfish (*Pangasianodon hypophthalmus*) genome. **Table S2.** Numbers of putative transcriptional regulator genes. **Table S3.** Numbers of genes encoding putative signaling molecules. **Table S4.** *Hox* genes in the striped catfish (*Pangasianodon hypophthalmus*) genome. **Table S5.** *IGFBP* genes used in molecular phylogenetic analysis. **Table S6.** The relationship between the striped catfish genome and channel catfish chromosomes. (DOCX 112 kb)

Additional file 2: **Figure S1.** Genome assembly, annotation, and validation pipeline in *Pangasianodon hypophthalmus*. **Figure S2.** Complete mitochondrial genome of striped catfish, *Pangasianodon hypophthalmus*. (PDF 864 kb)
