# Supplementary material for: A draft genome of the striped catfish, Pangasianodon hypophthalmus, for comparative analysis of genes relevant to development and a resource for aquaculture improvement
Source: BMC Genomics. 2018 Oct 5;19:733. doi: 10.1186/s12864-018-5079-x (PMC6173838; doi:10.1186/s12864-018-5079-x)
Supplement: Supplementary file 1 — Table S1. Summary of Miseq and Hiseq reads of striped catfish (Pangasianodon hypophthalmus) genome. Table S2. Numbers of putative transcriptional regulator genes. Table S3. Numbers of genes encoding putative signaling molecules. Table S4. Hox genes in the striped catfish (Pangasianodon hypophthalmus) genome. Table S5. IGFBP genes used in molecular phylogenetic analysis. Table S6. The relationship between the striped catfish genome and channel catfish chromosomes. (DOCX 112 kb) [file 12864_2018_5079_MOESM1_ESM.docx]

**Table S1. Summary of Miseq and Hiseq reads of striped catfish (*Pangasianodon hypophthalmus*) genome**

| Used data | | | | | | |  | Initial assembly | |
| --- | --- | --- | --- | --- | --- | --- | --- | --- | --- |
| Sequencer |  | Method | | Total sequences (Gbp) | Number of reads (million) | Average read length (bp) |  | Contig | Scaffold |
|  |  |  |  |  |  |  |  | N50 | N50 |
| Illumina |  | Paired-end shotgun | |  |  |  |  | 6 kbp | 8206 kbp |
|  |  |  | (MiSeq) | 28.47 | 92.63 | 310 |  |  |  |
|  |  |  | (Hiseq 2500) | 101.38 | 697.06 | 145 |  |  |  |
|  | Subtotal | |  | 129.85 | 789.69 |  |  |  |  |
|  |  |  |  |  |  |  |  |  |  |
|  |  | Mate pair (HiSeq 2500) | |  |  |  |  |  |  |
|  |  |  | 3kb | 50.35 | 176.71 | 295 |  |  |  |
|  |  |  | 7kb | 40.07 | 141.39 | 295 |  |  |  |
|  |  |  | 10kb | 86.98 | 304.19 | 295 |  |  |  |
|  |  |  | 15kb | 43.38 | 171.46 | 295 |  |  |  |
|  | Subtotal | |  | 220.78 | 793.75 |  |  |  |  |
|  | Total | |  | 350.63 | 1583.44 |  |  |  |  |

**Table S2. Numbers of putative transcriptional regulator genes**

| Accession | ID | Description | *O. latipes* | *T. rubripes* | *D. rerio* | *P. hypophthalmus* | *Ictalurus punctatus** |
| --- | --- | --- | --- | --- | --- | --- | --- |
| PF00010 | HLH | Helix-loop-helix DNA-binding domain | 140 | 300 | 209 | 152 | 300 |
| PF00046 | Homeobox | Homeobox domain | 284 | 657 | 455 | 290 | 525 |
| PF00096 | zf-C2H2 | Zinc finger, C2H2 type | 377 | 831 | 1602 | 349 | 1128 |
| PF00104 | Hormone_recep | Ligand-binding domain of nuclear hormone | 82 | 247 | 136 | 71 | 192 |
| PF00105 | zf-C4 | Zinc finger, C4 type (two domains) | 80 | 239 | 141 | 68 | 187 |
| PF00157 | Pou | Pou domain - N-terminal to homeobox domain | 18 | 55 | 23 | 19 | 37 |
| PF00170 | bZIP_1 | bZIP transcription factor | 80 | 205 | 113 | 78 | 162 |
| PF00178 | Ets | Ets-domain | 35 | 91 | 48 | 34 | 89 |
| PF00250 | Fork_head | Fork head domain | 50 | 137 | 95 | 62 | 111 |
| PF00292 | PAX | 'Paired box' domain | 27 | 54 | 40 | 11 | 46 |
| PF00319 | SRF-TF | SRF-type transcription factor | 9 | 41 | 19 | 7 | 29 |
| PF00320 | GATA | GATA zinc finger | 18 | 36 | 30 | 19 | 72 |
| PF00505 | HMG_box | HMG (high mobility group) box | 72 | 173 | 103 | 62 | 200 |
| PF00554 | RHD | Rel homology domain (RHD) | 11 | 20 | 15 | 14 | 38 |
| PF00751 | DM | DM DNA binding domain | 10 | 13 | 12 | 7 | 8 |
| PF00853 | Runt | Runt domain | 6 | 24 | 13 | 4 | 15 |
| PF00870 | P53 | P53 DNA-binding domain | 8 | 12 | 10 | 3 | 8 |
| PF00907 | T-box | T-box | 23 | 49 | 40 | 27 | 56 |
| PF01388 | ARID | ARID/BRIGHT DNA-binding domain | 16 | 52 | 35 | 20 | 41 |
| PF01530 | zf-C2HC | Zinc finger, C2HC type | 12 | 43 | 20 | 10 | 34 |
| PF01586 | Basic | Myogenic Basic domain | 4 | 12 | 5 | 4 | 5 |
| PF02023 | SCAN | SCAN domain | 3 | 3 | 0 | 6 | 13 |
| PF02178 | AT_hook | AT hook motif | 2 | 1 | 10 | 5 | 12 |
| PF02376 | CUT | CUT domain | 9 | 44 | 16 | 11 | 29 |
| PF03299 | TF_AP-2 | Transcription factor AP-2 | 6 | 9 | 13 | 6 | 15 |
| PF03529 | TF_Otx | Otx1 transcription factor | 6 | 7 | 6 | 6 | 9 |
| PF03615 | GCM | GCM motif protein | 1 | 2 | 3 | 1 | 1 |
| PF03826 | OAR | OAR domain | 22 | 32 | 29 | 20 | 32 |
| PF05044 | Prox1 | Homeo-prospero domain | 4 | 6 | 3 | 4 | 8 |
| PF06621 | SIM_C | Single-minded protein C-terminus | 2 | 4 | 3 | 2 | 8 |
| PF07527 | Hairy_orange | Hairy Orange | 16 | 33 | 26 | 31 | 33 |
| PF07710 | P53_tetramer | P53 tetramerisation motif | 8 | 12 | 10 | 5 | 8 |
| PF07716 | bZIP_2 | Basic region leucine zipper | 70 | 174 | 102 | 68 | 130 |
| PF12598 | TBX | T-box transcription factor | 2 | 4 | 4 | 7 | 8 |

*Data were obtained via ftp://ftp.ncbi.nlm.nih.gov/genomes/all/GCF/001/660/625/GCF_001660625.1_IpCoco_1.2/ and included 47974 proteins (GCF_001660625.1_IpCoco_1.2_protein.fa).

**Table S3. Numbers of genes encoding putative signaling molecules**

| Accession | ID | Description | *O. latipes* | *T. rubripes* | *D. rerio* | *P. hypophthalmus* | *Ictalurus punctatus* |
| --- | --- | --- | --- | --- | --- | --- | --- |
| PF00008 | EGF | EGF-like domain | 204 | 652 | 325 | 208 | 417 |
| PF00015 | MCPsignal | Methyl-accepting chemotaxis protein (MCP) signalling | 2 | 0 | 2 | 5 | 9 |
| PF00019 | TGF_beta | Transforming growth factor beta like | 51 | 101 | 57 | 49 | 67 |
| PF00049 | Insulin | Insulin/IGF/Relaxin family | 7 | 8 | 12 | 12 | 46 |
| PF00110 | wnt | wnt family | 29 | 55 | 42 | 27 | 59 |
| PF00167 | FGF | Fibroblast growth factor | 40 | 58 | 43 | 33 | 115 |
| PF00219 | IGFBP | Insulin-like growth factor binding protein | 34 | 115 | 37 | 35 | 0 |
| PF00341 | PDGF | PDGF/VEGF domain | 13 | 65 | 25 | 11 | 19 |
| PF00503 | G-alpha | G-protein alpha subunit | 77 | 103 | 94 | 71 | 115 |
| PF00615 | RGS | Regulator of G protein signaling | 62 | 175 | 71 | 43 | 99 |
| PF00631 | G-gamma | GGL domain | 15 | 36 | 28 | 18 | 28 |
| PF00672 | HAMP | HAMP domain | 0 | 1 | 1 | 0 | 46 |
| PF00688 | TGFb_propeptide | TGF-beta propeptide | 40 | 86 | 50 | 36 | 20 |
| PF00715 | IL2 | Interleukin 2 | 0 | 1 | 1 | 0 | 0 |
| PF00727 | IL4 | Interleukin 4 | 0 | 0 | 2 | 2 | 0 |
| PF00778 | DIX | DIX domain | 11 | 30 | 13 | 11 | 20 |
| PF01017 | STAT_alpha | STAT protein, all-alpha domain | 7 | 17 | 17 | 9 | 22 |
| PF01091 | PTN_MK_C | PTN/MK heparin-binding protein family, C-terminal | 6 | 8 | 4 | 3 | 5 |
| PF01415 | IL7 | Interleukin 7 | 0 | 1 | 0 | 1 | 0 |
| PF01534 | Frizzled | Frizzled/Smoothened family membrane region | 16 | 16 | 24 | 16 | 33 |
| PF01627 | Hpt | Hpt domain | 0 | 0 | 0 | 1 | 1 |
| PF02025 | IL5 | Interleukin 5 | 0 | 0 | 1 | 0 | 0 |
| PF02262 | Cbl_N | CBL proto-oncogene N-terminal domain 1 | 3 | 15 | 4 | 4 | 5 |
| PF02377 | Dishevelled | Dishevelled specific domain | 3 | 10 | 9 | 5 | 8 |
| PF02761 | Cbl_N2 | CBL proto-oncogene N-terminus, EF hand-like | 3 | 13 | 4 | 3 | 5 |
| PF02762 | Cbl_N3 | CBL proto-oncogene N-terminus, SH2-like domain | 2 | 13 | 4 | 3 | 5 |
| PF02864 | STAT_bind | STAT protein, DNA binding domain | 6 | 19 | 16 | 8 | 22 |
| PF02865 | STAT_int | STAT protein, protein interaction domain | 7 | 17 | 17 | 8 | 22 |
| PF03039 | IL12 | Interleukin 12 | 1 | 5 | 1 | 4 | 3 |
| PF03528 | Rabaptin | Rabaptin | 4 | 2 | 4 | 2 | 4 |
| PF03623 | Focal_AT | Focal adhesion targeting region | 4 | 22 | 7 | 4 | 29 |
| PF04692 | PDGF_N | Platelet-derived growth factor, N terminal | 6 | 18 | 7 | 1 | 5 |
| PF04709 | AMH_N | Anti-Mullerian hormone, N terminal region | 1 | 5 | 3 | 2 | 4 |
| PF05196 | PTN_MK_N | PTN/MK heparin-binding protein family, N-terminal | 5 | 6 | 4 | 3 | 5 |
| PF05337 | CSF-1 | Macrophage colony stimulating factor-1 | 0 | 0 | 2 | 2 | 3 |
| PF06554 | Olfactory_mark | Olfactory marker protein | 2 | 2 | 3 | 1 | 1 |
| PF07400 | IL11 | Interleukin 11 | 2 | 4 | 2 | 1 | 5 |
| PF07714 | Pkinase_Tyr | Tyrosine kinase | 716 | 2049 | 1385 | 635 | 0 |
| PF08916 | Phe_ZIP | Phenylalanine zipper | 2 | 8 | 4 | 3 | 6 |
| PF09034 | TRADD_N | TRADD, N-terminal domain | 1 | 1 | 1 | 1 | 2 |

No domains in *D. rerio* and *P. hypophthalmus* are omitted.

**Table S4. *Hox* genes in the striped catfish (*Pangasianodon hypophthalmus*) genome**

| Gene name | Query* | Scaffold number | Gene model ID*** | Transcriptome ID | Amino acid sequences from gene models of 2017**** |
| --- | --- | --- | --- | --- | --- |
| A1a | AEE90147.1 | 18 | phy_g9408.t1 | Not found |  |
| A2a | AEE90146.1 |  | Not found | Not found |  |
| A3a | AEE90145.1 | 18 | phy_g9407.t1 | s09_DN27450_c2_g2_i5 | |
| A4a | AEE90154.1 | 18 | phy_g9406.t1 | s02_DN14676_c0_g2_i1 | |
| A5a | AEE90153.1 |  | Not found | Not found |  |
| A7a | AEE90152.1 |  | Not found | Not found |  |
| A9a | AEE90151.1 | 18 | phy_g9405.t1 | s09_DN22659_c1_g4_i1 | |
| A10a | AEE90150.1 |  | Not found | Not found |  |
| A11a | AEE90149.1 |  | Not found | Not found |  |
| A13a | AEE90155.1 | 18 | phy_g9403.t1 | s01_DN41770_c0_g1_i1 | |
| A1b | AEE90162.1 |  | Not found | Not found |  |
| A2b | AEE90161.1 | 32 | phy_g7429.t1 | s11_DN24327_c2_g2_i2 | |
| A3b | AEE90160.1 |  | Not found | Not found |  |
| A9b | AEE90159.1 | 32 | phy_g7430.t1 | s08_DN17613_c0_g3_i1 | |
| A10b | AEE90158.1 | 32 | phy_g7431.t1 | s02_DN10581_c0_g1_i1 | |
| A11b | AEE90157.1 | 32 | phy_g7432.t1 | s10_DN30150_c1_g1_i1 | |
| A13b | AEE90156.1 | 32 | phy_g7434.t1 | s13_DN21122_c0_g1_i1 | |
| A13a | AEE90148.1 |  | Not found | Not found |  |
| B1a | AEE90173.1 | 6 | phy_g28212.t1 | s09_DN4777_c0_g1_i1 |  |
| B2a | AEE90172.1 | 6 | phy_g28210.t1 | s09_DN17140_c0_g1_i1 | |
| B3a | AEE90171.1 | 6 | phy_g28208.t1 | s10_DN28456_c0_g2_i3 | |
| B4a | AEE90170.1 | 6 | phy_g28207.t1 | s10_DN27504_c0_g1_i1 | |
| B5a | AEE90169.1 | 6 | phy_g28206.t1 | s02_DN25185_c1_g1_i1 | |
| B6a | AEE90168.1 | 6 | phy_g28205.t1 | s09_DN14259_c0_g1_i1 | |
| B7a | AEE90167.1 | 6 | phy_g28204.t1 | s09_DN14250_c0_g1_i1 | |
| B8a | AEE90166.1 | 6 | phy_g28203.t1 | s10_DN29156_c0_g1_i1 | |
| B9a | AEE90165.1 | 6 | phy_g28202.t1 | s09_DN16785_c0_g1_i5 | |
| B10a | AEE90164.1 |  | Not found | Not found |  |
| B13a | AEE90163.1 | 6 | phy_g28200.t1 | s01_DN20774_c0_g1_i1 | |
| B1b | AEE90182.1 | 5 | phy_g2717.t1 (g9435.t1) | s07_DN23907_c1_g2_i1 | g9435.t1 |
| B2b | AEE90181.1 |  | Not found | Not found |  |
| B3b | AEE90180.1 | 5 | phy_g2717.t1 (g9436.t1) | s07_DN23907_c1_g2_i1 | g9436.t1 |
| B4b | AEE90179.1 |  | Not found | Not found |  |
| B5b | AEE90178.1 | 5 | phy_g2718.t1 | s09_DN28660_c5_g2 |  |
| B6b | AEE90177.1 | 5 | phy_g2719.t1 | s09_DN28660_c5_g2_i3 | |
| B7b | AEE90176.1 |  | Not found | Not found |  |
| B8b | AEE90175.1 | 5 | phy_g2720.t1 (g9439.t1) | s09_DN21770_c0_g1 | g9439.t1 |
| B9b | AEE90174.1 |  | Not found | Not found |  |
| C1a | AEE90192.1 |  | phy_g1619.t1 | s20_DN23629_c0_g1_i1 | |
| C3a | NP_001128157.2** | | phy_g1620.t1 | s01_DN25361_c0_g2_i1 | |
| C4a | AEE90191.1 | 9 | phy_g1621.t1 | s11_DN27369_c2_g2_i1 | |
| C5a | AEE90190.1 | 9 | phy_g1622.t1 | s02_DN35065_c0_g2_i2 | |
| C6a | AEE90189.1 | 9 | phy_g1623.t1 | s08_DN23190_c0_g1_i1 | |
| C8a | AEE90188.1 | 9 | phy_g1624.t1 | s10_DN27474_c0_g1_i2 | |
| C9a | AEE90187.1 | 9 | phy_g1625.t1 | s09_DN21894_c0_g1_i1 | |
| C10a | AEE90186.1 | 9 | phy_g1626.t1 | s09_DN37597_c0_g1_i1 | |
| C11a | AEE90185.1 | 9 | phy_g1627.t1 | s09_DN8605_c0_g1_i1 |  |
| C12a | AEE90184.1 | 9 | phy_g1628.t1 | s02_DN22413_c0_g1_i1 | |
| C13a | AEE90183.1 | 9 | phy_g1629.t1 | s02_DN46628_c0_g1_i1 | |
| C4b | AEE90201.1 | 22 | phy_g27477.t1 | s12_DN24044_c0_g2_i1 | |
| C5b | AEE90200.1 | 22 | phy_g27478.t1 (g6497.t1) | s13_DN26449_c0_g1_i1 | g6497.t1 |
| C6b | AEE90199.1 | 22 | phy_g27478.t1 (g6498.t1) | s08_DN25699_c3_g4_i1 | g6498.t1 |
| C8b | AEE90198.1 |  | Not found | Not found |  |
| C9b | AEE90197.1 | 22 | phy_g27479.t1 | s09_DN330_c0_g2_i1 |  |
| C10b | AEE90196.1 |  | Not found | Not found |  |
| C11b | AEE90195.1 | 22 | phy_g27479.t2 | s10_DN28993_c0_g2_i5 | |
| C12b | AEE90194.1 | 22 | phy_g27480.t1 | s10_DN28993_c0_g3_i1 | |
| C13b | AEE90193.1 | 22 | phy_g27481.t1 | s11_DN15363_c1_g1_i1 | |
| D1a | AEE90210.1 | | phy_g8302.t1 | s10_DN24036_c1_g1_i1 | |
| D3a | AEE90209.1 | 3 | phy_g8303.t1 | s08_DN25658_c0_g2_i2 | |
| D4a | AEE90208.1 | 3 | phy_g8304.t1 | s08_DN25658_c0_g1_i2 | |
| D8a | AEE90207.1 | | Not found | Not found |  |
| D9a | AEE90206.1 | 3 | phy_g8305.t1 | s08_DN21599_c0_g1_i1 | |
| D10a | AEE90205.1 | 3 | phy_g8306.t1 | s09_DN38082_c0_g1_i1 | |
| D11a | AEE90204.1 | 3 | phy_g8307.t1 | s09_DN22745_c0_g1_i3 | |
| D12a | AEE90203.1 | 3 | (g19787.t1) | s10_DN30150_c0_g2_i1 | g19787.t1 |
| D13a | AEE90202.1 | 3 | phy_g8308.t1 | s10_DN20173_c0_g4_i1 | |
| D4b | AEE90215.1 |  | Not found | Not found |  |
| D9b | AEE90214.1 | | Not found | Not found |  |
| D10b | AEE90213.1 | | Not found | Not found |  |
| D11b | AEE90212.1 | | Not found | Not found |  |
| D12b | AEE90211.1 | | Not found | Not found |  |
| *From Henkel et al. (2012) for eel hox proteins | | | |  |  |
| **From Molven et al. (1992) for zebrafish hox protein | | | |  |  |
| ***IDs in parenthesis are from gene models of 2017. | | | |  |  |
| ****The better predicted sequences than those of 2018 are also shown. | | | | |  |

**Table S5. *IGFBP* genes used in molecular phylogenetic analysis**

| **Taxon name (alphabetical sorted)** | **Accession number** |
| --- | --- |
| *Acanthochromis polyacanthus* igfbp7 | XM_022218346.1 |
| *Amphiprion ocellaris* igfbp7 | XM_023277861.1 |
| *Cynoglossus semilaevis* igfbp5 variant X1 | XM_017038805.1 |
| *Cyprinodon variegatus* igfbp4 variant X1 | XM_015375833.1 |
| *Danio rerio* igfbp1 | NM_001098257.2 |
| *Danio rerio* igfbp1a | NM_173283.3 |
| *Danio rerio* igfbp2a | NM_131458.2 |
| *Danio rerio* igfbp3 | NM_205751.2 |
| *Danio rerio* igfbp5b | NM_001126463.1 |
| *Danio rerio* igfbp7 | NM_212924.2 |
| *Esox lucius* igfbp2 | XM_010893140.3 |
| *Esox lucius* igfbp4 variant X1 | XM_010889164.3 |
| *Haplochromis burtoni* igfbp2 | XM_005949761.2 |
| *Haplochromis burtoni* igfbp4 | XM_005919251.2 |
| *Haplochromis burtoni* igfbp7 | XM_005948654.2 |
| *Hippocampus comes* igfbp5 | XM_019856608.1 |
| *Homo sapiens* igfbp1 | NM_000596.3 |
| *Homo sapiens* IGFBP2 variant 1 | NM_000597.2 |
| *Homo sapiens* IGFBP3 variant 1 | NM_001013398.1 |
| *Homo sapiens* IGFBP4 | NM_001552.2 |
| *Homo sapiens* IGFBP5 | NM_000599.3 |
| *Homo sapiens* IGFBP6 | NM_002178.2 |
| *Homo sapiens* IGFBP7 variant 1 | NM_001553.2 |
| *Ictalurus punctatus* igfbp1 isoform X1 | XM_017472660.1 |
| *Ictalurus punctatus* igfbp2-A | XM_017482396.1 |
| *Ictalurus punctatus* igfbp2-B | XM_017469925.1 |
| *Ictalurus punctatus* igfbp3 | XM_017472659.1 |
| *Ictalurus punctatus* igfbp5 | XM_017469924.1 |
| *Ictalurus punctatus* igfbp5 variant2 | XM_017482450.1 |
| *Ictalurus punctatus* igfbp5 variant 3 | XM_017450722.1 |
| *Ictalurus punctatus* igfbp6 | XM_017487097.1 |
| *Ictalurus punctatus* igfbp7 | XM_017492053.1 |
| *Labrus bergylta* igfbp5 | XM_020636551.1 |
| *Labrus bergylta* igfbp6 | XM_020654898.1 |
| *Larimichthys crocea* igfbp3 | XM_019277499.1 |
| *Larimichthys crocea* igfbp4 | XM_010732633.2 |
| *Larimichthys crocea* igfbp6 | XM_010742620.2 |
| *Maylandia zebra* igfbp2 | XM_004562553.3 |
| *Maylandia zebra* igfbp4 | XM_004538727.4 |
| *Maylandia zebra* igfbp7 | XM_004563632.1 |
| *Mus musculus* igfbp1 | NM_008341.4 |
| *Mus musculus* Igfbp2 variant 1 | NM_008342.3 |
| *Mus musculus* Igfbp3 | NM_008343.2 |
| *Mus musculus* Igfbp4 | NM_010517.4 |
| *Mus musculus* Igfbp5 | NM_010518.2 |
| *Mus musculus* Igfbp6 | NM_008344.3 |
| *Mus musculus* Igfbp7 variant 2 | NM_008048.3 |
| *Nothobranchiusn furzeri* igfbp1 | KC306952.1 |
| *Nothobranchius furzeri* igfbp3 | KC306954.1 |
| *Nothobranchius furzeri* igfbp4 | KC306955.1 |
| *Nothobranchius furzeri* gfbp5 | KC306956.1 |
| *Nothobranchius furzeri* igfbp6 | KC306957.1 |
| *Oncorhynchus mykiss* igfbp1 | NM_001124561.1 |
| *Oncorhynchus mykiss* igfbp2 | NM_001124649.1 |
| *Oncorhynchus mykiss* igfbp3 | NM_001124557.1 |
| *Oncorhynchus mykiss* igfbp5 | NM_001124652.1 |
| *Oncorhynchus mykiss* igfbp6 | NM_001124560.1 |
| *Oncorhynchus mykiss* igfbp7 | NM_001124648.3 |
| *Oreochromis niloticus* igfbp2 variant X1 | XM_003453175.4 |
| *Oreochromis niloticus* igfbp4 | XM_003454633.4 |
| *Oreochromis niloticus* igfbp7 | XM_003458662.4 |
| *Oryzias latipes* igfbp2 variant X1 | XM_023964915.1 |
| *Oryzias latipes* igfbp4 variant X1 | XM_023955145.1 |
| *Pangasianodon hypophthalmus* g11973.t1 | This study |
| *Pangasianodon hypophthalmus* g11974.t1 | This study |
| *Pangasianodon hypophthalmus* g1604.t1 | This study |
| *Pangasianodon hypophthalmus* g24144.t1 | This study |
| *Pangasianodon hypophthalmus* g24145.t1 | This study |
| *Pangasianodon hypophthalmus* g24954.t1 | This study |
| *Pangasianodon hypophthalmus* g27470.t1 | This study |
| *Pangasianodon hypophthalmus* g8120.t1 | This study |
| *Pangasianodon hypophthalmus* g8121.t1 | This study |
| *Pangasianodon hypophthalmus* g8121.t2 | This study |
| *Pangasianodon hypophthalmus* g8896.t1 | This study |
| *Pangasianodon hypophthalmus* g8897.t1 | This study |
| *Paralichthys olivaceus* IGFBP2 | KC914560.1 |
| *Paralichthys olivaceus* Igfbp2b | KC914561.1 |
| *Paralichthys olivaceus* igfbp5 | XM_020099752.1 |
| *Poecilia formosa* igfbp1 | XM_007559352.2 |
| *Poecilia formosa* igfbp2 variant X1 | XM_007567581.2 |
| *Poecilia formosa* igfbp4 variant X1 | XM_007567306.2 |
| *Poecilia latipinna* igfbp4 variant X1 | XM_015039673.1 |
| *Poecilia mexicana* igfbp1 | XM_015000519.1 |
| *Poecilia mexicana* igfbp2 variant X1 | XM_014990518.1 |
| *Poecilia mexicana* igfbp4 variant X1 | XM_014988182.1 |
| *Poecilia reticulata* igfbp2 variant X1 | XM_008423480.2 |
| *Poecilia reticulata* igfbp3 | XM_008406723.2 |
| *Poecilia reticulata* igfbp4 variant X1 | XM_008406547.2 |
| *Poecilia reticulata* igfbp6 variant X1 | XM_008414626.2 |
| *Pundamilia nyererei* igfbp2 | XM_005753098.1 |
| *Pundamilia nyererei* igfbp4 | XM_005746028.1 |
| *Pundamilia nyererei* igfbp7 | XM_005719381.2 |
| *Pygocentrus nattereri* igfbp2 | XM_017691593.1 |
| *Salmo salar* igfbp1 | EF432856.1 |
| *Salmo salar* igfbp2 | EF432858.1 |
| *Salmo salar* igfbp3 (igfbp-2b1) | NM_001123648.1 |
| *Salmo salar* igfbp5 | EF432862.1 |
| *Salmo salar* igfbp6 | NM_001123650.1 |
| *Salvelinus alpinus* igfbp1 | GU933433.1 |
| *Salvelinus alpinus* igfbp2b | GU933432.1 |
| *Seriola dumerili* igfbp4 variant X1 | XM_022739957.1 |
| *Seriola dumerili* igfbp7 | XM_022761348.1 |
| *Seriola lalandi dorsalis* igfbp4 | XM_023413676.1 |
| *Seriola lalandi dorsalis* igfbp6 | XM_023392914.1 |
| *Seriolan lalandi dorsalis* igfbp7 | XM_023420689.1 |
| *Sinocyclocheilus grahami* igfbp2 | XM_016273640.1 |
| *Tachysurus fulvidraco* igfbp3 | KT895993.1 |
| *Tachysurus fulvidraco* igfbp5 | KT895994.1 |
| *Takifugu rubripes* igfbp1 | XM_011616123.1 |
| *Takifugu rubripes* igfbp2 | XM_003966591.2 |
| *Takifugu rubripes* igfbp3 variant X1 | XM_003975304.2 |
| *Takifugu rubripes* igfbp4 | NM_001146062.1 |
| *Takifugu rubripes* igfbp6 | XM_003976104.2 |
| *Xiphophorus maculatus* igfbp2 variant X1 | XM_023329952.1 |
| *Xiphophorus maculatus* igfbp3 | XM_005802306.3 |

**Table S6. The relationship between the striped catfish genome and channel catfish chromosomes**

| Scaffold of striped catfish | Scaffold length of striped catfish (bp) | Gene number on scaffold of striped catfish | Maximum hits for a chromosome of channel catfish (gene number) | Best hit chromosome to channel catfish (NCBI_ID) |
| --- | --- | --- | --- | --- |
| sc0000001 | 37494740 | 2587 | 604 | NC_030426.1 |
| sc0000002 | 23786125 | 1601 | 967 | NC_030418.1 |
| sc0000003 | 23232829 | 1519 | 912 | NC_030421.1 |
| sc0000004 | 23204775 | 1516 | 440 | NC_030419.1 |
| sc0000005 | 20940362 | 1371 | 959 | NC_030428.1 |
| sc0000006 | 20782094 | 1598 | 923 | NC_030417.1 |
| sc0000007 | 19917768 | 1605 | 484 | NC_030423.1 |
| sc0000008 | 21676741 | 1524 | 851 | NC_030429.1 |
| sc0000009 | 18849081 | 1408 | 912 | NC_030430.1 |
| sc0000010 | 17209216 | 1309 | 771 | NC_030443.1 |
| sc0000011 | 23808341 | 1450 | 628 | NC_030434.1 |
| sc0000012 | 16899263 | 1228 | 389 | NC_030438.1 |
| sc0000013 | 15945287 | 1238 | 351 | NC_030420.1 |
| sc0000014 | 15842060 | 1208 | 468 | NC_030416.1 |
| sc0000015 | 16210006 | 1130 | 619 | NC_030419.1 |
| sc0000016 | 14685745 | 1176 | 329 | NC_030420.1 |
| sc0000017 | 14288580 | 988 | 399 | NC_030433.1 |
| sc0000018 | 13895861 | 929 | 647 | NC_030439.1 |
| sc0000019 | 12970507 | 907 | 622 | NC_030425.1 |
| sc0000020 | 12363827 | 738 | 545 | NC_030437.1 |
| sc0000021 | 12030981 | 906 | 566 | NC_030441.1 |
| sc0000022 | 11696205 | 863 | 422 | NC_030436.1 |
| sc0000023 | 11526600 | 816 | 431 | NC_030427.1 |
| sc0000024 | 11033978 | 724 | 577 | NC_030424.1 |
| sc0000025 | 10842290 | 789 | 426 | NC_030432.1 |
| sc0000026 | 10743009 | 958 | 432 | NC_030436.1 |
| sc0000027 | 21807806 | 1652 | 1037 | NC_030422.1 |
| sc0000028 | 10517856 | 588 | 421 | NC_030442.1 |
| sc0000029 | 10401864 | 789 | 441 | NC_030438.1 |
| sc0000030 | 10126641 | 752 | 549 | NC_030433.1 |
| sc0000031 | 9983477 | 733 | 379 | NC_030418.1 |
| sc0000032 | 9596357 | 818 | 281 | NC_030416.1 |
| sc0000033 | 8913854 | 713 | 319 | NC_030423.1 |
| sc0000034 | 13330545 | 1027 | 436 | NC_030435.1 |
| sc0000035 | 7783948 | 554 | 292 | NC_030431.1 |
| sc0000036 | 7468867 | 444 | 300 | NC_030427.1 |
| sc0000037 | 7623426 | 568 | 346 | NC_030431.1 |
| sc0000038 | 6972118 | 627 | 256 | NC_030444.1 |
| sc0000039 | 6787112 | 601 | 200 | NC_030432.1 |
| sc0000040 | 6637728 | 549 | 336 | NC_030431.1 |
| sc0000041 | 6100290 | 352 | 212 | NC_030424.1 |
| sc0000042 | 6066629 | 368 | 258 | NC_030440.1 |
| sc0000043 | 5969716 | 332 | 197 | NC_030416.1 |
| sc0000044 | 5158973 | 400 | 224 | NC_030430.1 |
| sc0000045 | 4873072 | 294 | 192 | NC_030444.1 |
| sc0000046 | 4792111 | 467 | 213 | NC_030426.1 |
| sc0000047 | 4049363 | 394 | 222 | NC_030420.1 |
| sc0000048 | 3963117 | 372 | 235 | NC_030439.1 |
| sc0000049 | 3575745 | 294 | 211 | NC_030435.1 |
| sc0000050 | 3095902 | 207 | 136 | NC_030423.1 |
| sc0000051 | 2789637 | 228 | 150 | NC_030423.1 |
| sc0000052 | 2700707 | 272 | 101 | NC_030442.1 |
| sc0000053 | 2621943 | 196 | 121 | NC_030440.1 |
| sc0000054 | 2620060 | 218 | 159 | NC_030417.1 |
| sc0000055 | 2548504 | 240 | 58 | NC_030421.1 |
| sc0000056 | 2536578 | 174 | 99 | NC_030429.1 |
| sc0000057 | 2374167 | 171 | 82 | NC_030423.1 |
| sc0000058 | 2359716 | 251 | 130 | NC_030422.1 |
| sc0000059 | 2181210 | 182 | 99 | NC_030444.1 |
| sc0000060 | 1833706 | 157 | 72 | NC_030442.1 |
| sc0000061 | 1753796 | 227 | 94 | NC_030428.1 |
| sc0000062 | 1645754 | 118 | 61 | NC_030416.1 |
| sc0000063 | 1614577 | 154 | 91 | NC_030442.1 |
| sc0000064 | 1492446 | 148 | 85 | NC_030440.1 |
| sc0000065 | 1369170 | 144 | 51 | NC_030424.1 |
| sc0000066 | 1291818 | 134 | 62 | NC_030427.1 |
| sc0000067 | 1277760 | 160 | 14 | NC_030431.1 |
| sc0000068 | 1258165 | 123 | 75 | NC_030417.1 |
| sc0000069 | 1249612 | 127 | 49 | NC_030429.1 |
| sc0000070 | 1197254 | 109 | 39 | NC_030434.1 |
| sc0000071 | 1392908 | 157 | 42 | NC_030425.1 |
| sc0000072 | 1149405 | 130 | 41 | NC_030427.1 |
| sc0000073 | 1135668 | 137 | 41 | NC_030422.1 |
| sc0000074 | 1119071 | 166 | 65 | NC_030427.1 |
| sc0000075 | 1094570 | 143 | 70 | NC_030422.1 |
| sc0000076 | 1049178 | 106 | 79 | NC_030431.1 |
| sc0000077 | 1032698 | 92 | 30 | NC_030443.1 |
| sc0000078 | 1555068 | 155 | 87 | NC_030442.1 |
| sc0000079 | 1022846 | 145 | 59 | NC_030421.1 |
| sc0000080 | 963568 | 104 | 58 | NC_030432.1 |
| sc0000081 | 896575 | 77 | 33 | NC_030423.1 |
| sc0000082 | 823351 | 133 | 20 | NC_030439.1 |
| sc0000083 | 768645 | 66 | 36 | NC_030420.1 |
| sc0000084 | 755361 | 98 | 50 | NC_030444.1 |
| sc0000085 | 716149 | 93 | 17 | NC_030427.1 |
| sc0000086 | 608299 | 58 | 35 | NC_030433.1 |
| sc0000087 | 591339 | 79 | 34 | NC_030418.1 |
| sc0000088 | 574980 | 64 | 21 | NC_030416.1 |
| sc0000089 | 554450 | 60 | 20 | NC_030426.1 |
| sc0000090 | 541608 | 43 | 17 | NC_030434.1 |
| sc0000091 | 641633 | 85 | 29 | NC_030418.1 |
| sc0000092 | 484685 | 61 | 15 | NC_030423.1 |
| sc0000093 | 761946 | 130 | 48 | NC_030440.1 |
| sc0000094 | 438320 | 45 | 27 | NC_030438.1 |
| sc0000095 | 434512 | 70 | 17 | NC_030440.1 |
| sc0000096 | 431626 | 82 | 30 | NC_030441.1 |
| sc0000097 | 402139 | 39 | 27 | NC_030428.1 |
| sc0000098 | 495690 | 57 | 17 | NC_030419.1 |
| sc0000099 | 391953 | 62 | 25 | NC_030441.1 |
| sc0000100 | 390202 | 51 | 16 | NC_030429.1 |
| sc0000101 | 381976 | 36 | 9 | NC_030437.1 |
| sc0000102 | 366000 | 31 | 21 | NC_030437.1 |
| sc0000103 | 356860 | 29 | 21 | NC_030435.1 |
| sc0000104 | 347956 | 33 | 24 | NC_030437.1 |
| sc0000105 | 417424 | 45 | 30 | NC_030417.1 |
| sc0000106 | 322974 | 29 | 14 | NC_030424.1 |
| sc0000107 | 336106 | 44 | 19 | NC_030440.1 |
| sc0000108 | 309243 | 30 | 13 | NC_030444.1 |
| sc0000109 | 308169 | 25 | 16 | NC_030438.1 |
| sc0000110 | 293744 | 48 | 14 | NC_030427.1 |
| sc0000111 | 285154 | 41 | 13 | NC_030429.1 |
| sc0000112 | 303904 | 38 | 12 | NC_030437.1 |
| sc0000113 | 356289 | 50 | 8 | NC_030442.1 |
| sc0000114 | 256706 | 22 | 11 | NC_030420.1 |
| sc0000115 | 253695 | 39 | 18 | NC_030416.1 |
| sc0000116 | 252461 | 24 | 16 | NC_030430.1 |
| sc0000117 | 245317 | 24 | 9 | NC_030418.1 |
| sc0000118 | 250771 | 17 | 12 | NC_030424.1 |
| sc0000119 | 235538 | 24 | 20 | NC_030417.1 |
| sc0000120 | 230009 | 30 | 12 | NC_030416.1 |
| sc0000121 | 217961 | 33 | 14 | NC_030417.1 |
| sc0000122 | 222963 | 35 | 7 | NC_030434.1 |
| sc0000123 | 211625 | 30 | 12 | NC_030429.1 |
| sc0000124 | 208160 | 45 | 4 | NC_030435.1 |
| sc0000125 | 198703 | 21 | 11 | NC_030435.1 |
| sc0000126 | 199141 | 25 | 10 | NC_030422.1 |
| sc0000127 | 195216 | 32 | 12 | NC_030417.1 |
| sc0000128 | 189043 | 23 | 17 | NC_030430.1 |
| sc0000129 | 180369 | 18 | 7 | NC_030416.1 |
| sc0000130 | 178133 | 19 | 13 | NC_030430.1 |
| sc0000131 | 171830 | 19 | 11 | NC_030431.1 |
| sc0000132 | 180153 | 35 | 9 | NC_030427.1 |
| sc0000133 | 169418 | 17 | 11 | NC_030442.1 |
| sc0000134 | 171042 | 21 | 10 | NC_030432.1 |
| sc0000135 | 169577 | 32 | 6 | NC_030429.1 |
| sc0000136 | 166126 | 14 | 7 | NC_030430.1 |
| sc0000137 | 180862 | 17 | 10 | NC_030435.1 |
| sc0000138 | 151398 | 14 | 11 | NC_030424.1 |
| sc0000139 | 154543 | 25 | 3 | NC_030425.1 |
| sc0000140 | 147934 | 21 | 5 | NC_030424.1 |
| sc0000141 | 136184 | 14 | 10 | NC_030433.1 |
| sc0000142 | 135423 | 15 | 10 | NC_030421.1 |
| sc0000143 | 132110 | 20 | 12 | NC_030423.1 |
| sc0000144 | 133316 | 18 | 15 | NC_030430.1 |
| sc0000145 | 131720 | 8 | 4 | NC_030420.1 |
| sc0000146 | 124868 | 9 | 6 | NC_030419.1 |
| sc0000147 | 119086 | 9 | 7 | NC_030444.1 |
| sc0000148 | 122577 | 19 | 10 | NC_030416.1 |
| sc0000149 | 212735 | 33 | 4 | NC_030418.1 |
| sc0000150 | 122388 | 22 | 7 | NC_030422.1 |
| sc0000151 | 120850 | 17 | 8 | NC_030443.1 |
| sc0000152 | 108936 | 16 | 7 | NC_030436.1 |
| sc0000153 | 108971 | 8 | 6 | NC_030420.1 |
| sc0000154 | 99682 | 8 | 6 | NC_030420.1 |
| sc0000155 | 99178 | 5 | 1 | NC_030433.1 |
| sc0000156 | 99150 | 10 | 8 | NC_030431.1 |
| sc0000157 | 96899 | 7 | 6 | NC_030424.1 |
| sc0000158 | 96364 | 8 | 7 | NC_030437.1 |
| sc0000159 | 104908 | 13 | 6 | NC_030420.1 |
| sc0000160 | 94484 | 10 | 5 | NC_030422.1 |
| sc0000161 | 91933 | 10 | 4 | NC_030444.1 |
| sc0000162 | 84850 | 11 | 3 | NC_030432.1 |
| sc0000163 | 94523 | 12 | 6 | NC_030419.1 |
| sc0000164 | 84774 | 9 | 6 | NC_030432.1 |
| sc0000165 | 84579 | 7 | 4 | NC_030444.1 |
| sc0000166 | 83896 | 7 | 7 | NC_030427.1 |
| sc0000167 | 79893 | 11 | 6 | NC_030440.1 |
| sc0000168 | 77202 | 6 | 5 | NC_030423.1 |
| sc0000169 | 75201 | 8 | 1 | NC_030444.1 |
| sc0000170 | 75464 | 26 | 3 | NC_030440.1 |
| sc0000171 | 70945 | 11 | 2 | NC_030434.1 |
| sc0000172 | 70245 | 8 | 7 | NC_030430.1 |
| sc0000173 | 69880 | 9 | 5 | NC_030422.1 |
| sc0000174 | 70599 | 8 | 4 | NC_030418.1 |
| sc0000175 | 66315 | 8 | 5 | NC_030417.1 |
| sc0000176 | 65332 | 7 | 4 | NC_030434.1 |
| sc0000177 | 72983 | 4 | 2 | NC_030441.1 |
| sc0000178 | 58862 | 4 | 4 | NC_030431.1 |
| sc0000179 | 56719 | 4 | 4 | NC_030431.1 |
| sc0000180 | 94219 | 14 | 4 | NC_030441.1 |
| sc0000181 | 56674 | 4 | 4 | NC_030417.1 |
| sc0000182 | 57784 | 15 | 1 | NC_030440.1 |
| sc0000183 | 58006 | 8 | 7 | NC_030436.1 |
| sc0000184 | 55110 | 7 | 3 | NC_030424.1 |
| sc0000185 | 53584 | 8 | 7 | NC_030443.1 |
| sc0000186 | 47715 | 9 | 6 | NC_030441.1 |
| sc0000187 | 47993 | 2 | 2 | NC_030436.1 |
| sc0000188 | 48256 | 7 | 2 | NC_030437.1 |
| sc0000189 | 47430 | 2 | 1 | NC_030420.1 |
| sc0000190 | 43519 | 4 | 4 | NC_030443.1 |
| sc0000191 | 49865 | 12 | 1 | NC_030442.1 |
| sc0000192 | 39454 | 9 | 6 | NC_030437.1 |
| sc0000193 | 39363 | 6 | 2 | NC_030429.1 |
| sc0000194 | 38952 | 11 | 1 | NC_030443.1 |
| sc0000195 | 38299 | 3 | 2 | NC_030417.1 |
| sc0000196 | 62276 | 13 | 4 | NC_030443.1 |
| sc0000197 | 33716 | 4 | 4 | NC_030443.1 |
| sc0000198 | 33176 | 4 | 4 | NC_030443.1 |
| sc0000199 | 34383 | 6 | 2 | NC_030418.1 |
| sc0000200 | 33654 | 6 | 2 | NC_030420.1 |
| sc0000201 | 32448 | 2 | 2 | NC_030420.1 |
| sc0000202 | 31620 | 6 | 3 | NC_030444.1 |
| sc0000203 | 32160 | 4 | 2 | NC_030428.1 |
| sc0000204 | 31142 | 5 | 4 | NC_030433.1 |
| sc0000205 | 31517 | 7 | 3 | NC_030444.1 |
| sc0000206 | 29170 | 2 | 1 | NC_030439.1 |
| sc0000207 | 27180 | 6 | 3 | NC_030434.1 |
| sc0000208 | 28404 | 3 | 3 | NC_030444.1 |
| sc0000209 | 41200 | 4 | 2 | NC_030437.1 |
| sc0000210 | 28462 | 1 | 1 | NC_030431.1 |
| sc0000211 | 28241 | 3 | 3 | NC_030437.1 |
| sc0000212 | 26393 | 5 | 1 | NC_030420.1 |
| sc0000213 | 25810 | 1 | 1 | NC_030423.1 |
| sc0000215 | 20321 | 2 | 2 | NC_030436.1 |
| sc0000216 | 20723 | 2 | 1 | NC_030422.1 |
| sc0000217 | 29372 | 4 | 3 | NC_030424.1 |
| sc0000218 | 37066 | 11 | 1 | NC_030435.1 |
| sc0000219 | 19550 | 4 | 1 | NC_030437.1 |
| sc0000220 | 17990 | 4 | 1 | NC_030437.1 |
| sc0000221 | 33428 | 4 | 4 | NC_030424.1 |
| sc0000222 | 17529 | 3 | 1 | NC_030431.1 |
| sc0000224 | 16030 | 1 | 1 | NC_030420.1 |
| sc0000225 | 16266 | 3 | 2 | NC_030423.1 |
| sc0000226 | 15130 | 3 | 1 | NC_030435.1 |
| sc0000227 | 14473 | 2 | 1 | NC_030417.1 |
| sc0000228 | 14099 | 7 | 1 | NC_030444.1 |
| sc0000229 | 14330 | 2 | 1 | NC_030427.1 |
| sc0000231 | 12164 | 2 | 1 | NC_030416.1 |
| sc0000232 | 12109 | 3 | 2 | NC_030425.1 |
| sc0000234 | 11668 | 4 | 3 | NC_030430.1 |
| sc0000235 | 10836 | 3 | 1 | NC_030442.1 |
| sc0000237 | 10339 | 2 | 2 | NC_030441.1 |
| sc0000238 | 10225 | 4 | 2 | NC_030437.1 |
| sc0000239 | 9876 | 2 | 2 | NC_030443.1 |
| sc0000240 | 9558 | 3 | 2 | NC_030438.1 |
| sc0000241 | 8663 | 2 | 2 | NC_030425.1 |
| sc0000243 | 7741 | 1 | 1 | NC_030442.1 |
| sc0000244 | 6518 | 1 | 1 | NC_030420.1 |
| sc0000246 | 7650 | 2 | 2 | NC_030417.1 |
| sc0000248 | 7475 | 1 | 1 | NC_030417.1 |
| sc0000250 | 6699 | 1 | 1 | NC_030440.1 |
| sc0000256 | 5809 | 1 | 1 | NC_030442.1 |
| sc0000257 | 5974 | 1 | 1 | NC_030425.1 |
| sc0000258 | 5688 | 1 | 1 | NC_030436.1 |
| sc0000259 | 5377 | 1 | 1 | NC_030442.1 |
| sc0000260 | 5401 | 1 | 1 | NC_030442.1 |
| sc0000262 | 5318 | 2 | 2 | NC_030424.1 |
| sc0000263 | 5136 | 2 | 1 | NC_030443.1 |
| sc0000266 | 4960 | 1 | 1 | NC_030437.1 |
| sc0000267 | 5134 | 1 | 1 | NC_030443.1 |
| sc0000268 | 4942 | 1 | 1 | NC_030416.1 |
| sc0000273 | 4504 | 2 | 1 | NC_030435.1 |
| sc0000274 | 4456 | 1 | 1 | NC_030442.1 |
| sc0000279 | 4136 | 1 | 1 | NC_030437.1 |
| sc0000285 | 4084 | 1 | 1 | NC_030441.1 |
| sc0000287 | 4134 | 1 | 1 | NC_030439.1 |
| sc0000288 | 4056 | 2 | 1 | NC_030440.1 |
| sc0000289 | 3956 | 2 | 2 | NC_030428.1 |
| sc0000291 | 4237 | 2 | 1 | NC_030438.1 |
| sc0000292 | 3891 | 1 | 1 | NC_030443.1 |
| sc0000296 | 3759 | 1 | 1 | NC_030443.1 |
| sc0000297 | 3751 | 1 | 1 | NC_030427.1 |
| sc0000301 | 3582 | 1 | 1 | NC_030419.1 |
| sc0000302 | 3571 | 1 | 1 | NC_030417.1 |
| sc0000303 | 3515 | 1 | 1 | NC_030429.1 |
| sc0000305 | 3368 | 1 | 1 | NC_030427.1 |
| sc0000306 | 3335 | 2 | 2 | NC_030443.1 |
| sc0000307 | 19152 | 1 | 1 | NC_030443.1 |
| sc0000308 | 3402 | 1 | 1 | NC_030427.1 |
| sc0000311 | 3083 | 1 | 1 | NC_030442.1 |
| sc0000312 | 3060 | 1 | 1 | NC_030425.1 |
| sc0000314 | 3050 | 1 | 1 | NC_030424.1 |
| sc0000315 | 3023 | 1 | 1 | NC_030423.1 |
| sc0000317 | 2950 | 1 | 1 | NC_030422.1 |
| sc0000319 | 2930 | 1 | 1 | NC_030419.1 |
| sc0000323 | 2884 | 1 | 1 | NC_030443.1 |
| sc0000325 | 2829 | 2 | 1 | NC_030443.1 |
| sc0000326 | 17566 | 2 | 1 | NC_030418.1 |
| sc0000328 | 2780 | 1 | 1 | NC_030437.1 |
| sc0000331 | 2623 | 1 | 1 | NC_030431.1 |
| sc0000333 | 2547 | 1 | 1 | NC_030420.1 |
| sc0000334 | 2536 | 1 | 1 | NC_030442.1 |
| sc0000335 | 2499 | 2 | 2 | NC_030423.1 |
| sc0000336 | 2484 | 1 | 1 | NC_030432.1 |
| sc0000338 | 2458 | 1 | 1 | NC_030435.1 |
| sc0000344 | 2348 | 1 | 1 | NC_030430.1 |
| sc0000346 | 2421 | 1 | 1 | NC_030436.1 |
| sc0000347 | 2308 | 1 | 1 | NC_030437.1 |
| sc0000348 | 2299 | 1 | 1 | NC_030435.1 |
| sc0000350 | 2290 | 1 | 1 | NC_030434.1 |
| sc0000352 | 2270 | 1 | 1 | NC_030429.1 |
| sc0000353 | 2270 | 1 | 1 | NC_030424.1 |
| sc0000357 | 2258 | 1 | 1 | NC_030430.1 |
| sc0000359 | 2253 | 1 | 1 | NC_030420.1 |
| sc0000361 | 2244 | 2 | 1 | NC_030416.1 |
| sc0000362 | 2225 | 1 | 1 | NC_030437.1 |
| sc0000366 | 2209 | 1 | 1 | NC_030421.1 |
| sc0000367 | 2206 | 2 | 1 | NC_030428.1 |
| sc0000368 | 2201 | 1 | 1 | NC_030427.1 |
| sc0000369 | 2574 | 1 | 1 | NC_030440.1 |
| sc0000372 | 2181 | 1 | 1 | NC_030424.1 |
| sc0000373 | 2179 | 1 | 1 | NC_030428.1 |
| sc0000376 | 2159 | 1 | 1 | NC_030424.1 |
| sc0000377 | 2152 | 1 | 1 | NC_030435.1 |
| sc0000378 | 2147 | 1 | 1 | NC_030432.1 |
| sc0000379 | 2144 | 1 | 1 | NC_030430.1 |
| sc0000382 | 2120 | 1 | 1 | NC_030443.1 |
| sc0000387 | 2103 | 1 | 1 | NC_030441.1 |
| sc0000388 | 2098 | 2 | 1 | NC_030422.1 |
| sc0000390 | 2096 | 1 | 1 | NC_030422.1 |
| sc0000391 | 2093 | 1 | 1 | NC_030426.1 |
| sc0000395 | 2070 | 1 | 1 | NC_030419.1 |
| sc0000396 | 2066 | 1 | 1 | NC_030438.1 |
| sc0000398 | 2045 | 1 | 1 | NC_030417.1 |
| sc0000407 | 2012 | 1 | 1 | NC_030436.1 |
| sc0000408 | 2010 | 1 | 1 | NC_030428.1 |
| sc0000417 | 1960 | 1 | 1 | NC_030432.1 |
| sc0000420 | 1953 | 1 | 1 | NC_030437.1 |
| sc0000422 | 1939 | 1 | 1 | NC_030443.1 |
| sc0000424 | 1922 | 1 | 1 | NC_030417.1 |
| sc0000425 | 1921 | 1 | 1 | NC_030436.1 |
| sc0000426 | 1912 | 2 | 1 | NC_030417.1 |
| sc0000428 | 1905 | 2 | 1 | NC_030430.1 |
| sc0000431 | 1893 | 1 | 1 | NC_030426.1 |
| sc0000432 | 1891 | 1 | 1 | NC_030417.1 |
| sc0000436 | 1874 | 1 | 1 | NC_030416.1 |
| sc0000437 | 1874 | 1 | 1 | NC_030425.1 |
| sc0000440 | 1859 | 1 | 1 | NC_030444.1 |
| sc0000443 | 1847 | 1 | 1 | NC_030418.1 |
| sc0000444 | 1846 | 1 | 1 | NC_030431.1 |
| sc0000450 | 1829 | 1 | 1 | NC_030444.1 |
| sc0000452 | 1819 | 1 | 1 | NC_030443.1 |
| sc0000453 | 1818 | 1 | 1 | NC_030419.1 |
| sc0000454 | 1807 | 1 | 1 | NC_030419.1 |
| sc0000456 | 1794 | 1 | 1 | NC_030442.1 |
| sc0000458 | 1775 | 2 | 1 | NC_030434.1 |
| sc0000460 | 1769 | 1 | 1 | NC_030419.1 |
| sc0000463 | 1762 | 1 | 1 | NC_030437.1 |
| sc0000469 | 1750 | 1 | 1 | NC_030435.1 |
| sc0000471 | 1749 | 1 | 1 | NC_030436.1 |
| sc0000472 | 1747 | 1 | 1 | NC_030436.1 |
| sc0000486 | 1705 | 1 | 1 | NC_030430.1 |
| sc0000492 | 1690 | 1 | 1 | NC_030430.1 |
| sc0000496 | 1681 | 1 | 1 | NC_030428.1 |
| sc0000500 | 1677 | 1 | 1 | NC_030417.1 |
| sc0000501 | 1676 | 1 | 1 | NC_030439.1 |
| sc0000502 | 1673 | 1 | 1 | NC_030435.1 |
| sc0000504 | 1664 | 1 | 1 | NC_030441.1 |
| sc0000505 | 1663 | 2 | 1 | NC_030429.1 |
| sc0000507 | 1660 | 1 | 1 | NC_030435.1 |
| sc0000508 | 1659 | 1 | 1 | NC_030443.1 |
| sc0000519 | 1637 | 1 | 1 | NC_030424.1 |
| sc0000524 | 1623 | 2 | 1 | NC_030437.1 |
| sc0000527 | 1606 | 1 | 1 | NC_030424.1 |
| sc0000528 | 1604 | 1 | 1 | NC_030437.1 |
| sc0000529 | 1604 | 1 | 1 | NC_030430.1 |
| sc0000532 | 1581 | 1 | 1 | NC_030420.1 |
| sc0000534 | 1579 | 1 | 1 | NC_030420.1 |
| sc0000537 | 1569 | 1 | 1 | NC_030417.1 |
| sc0000542 | 1546 | 1 | 1 | NC_030417.1 |
| sc0000543 | 1544 | 1 | 1 | NC_030423.1 |
| sc0000546 | 1537 | 1 | 1 | NC_030437.1 |
| sc0000547 | 1534 | 1 | 1 | NC_030444.1 |
| sc0000550 | 1524 | 1 | 1 | NC_030426.1 |
| sc0000560 | 1503 | 1 | 1 | NC_030420.1 |
| sc0000566 | 750 | 1 | 1 | NC_030418.1 |
| sc0000568 | 592 | 1 | 1 | NC_030439.1 |
|  |  |  |  |  |
