# Supplementary material for: A draft genome of the striped catfish, Pangasianodon hypophthalmus, for comparative analysis of genes relevant to development and a resource for aquaculture improvement
Source: BMC Genomics. 2018 Oct 5;19:733. doi: 10.1186/s12864-018-5079-x (PMC6173838; doi:10.1186/s12864-018-5079-x)
Supplement: Supplementary file 2 — Figure S1. Genome assembly, annotation, and validation pipeline in Pangasianodon hypophthalmus. Figure S2. Complete mitochondrial genome of striped catfish, Pangasianodon hypophthalmus. (PDF 864 kb) [file 12864_2018_5079_MOESM2_ESM.pdf]

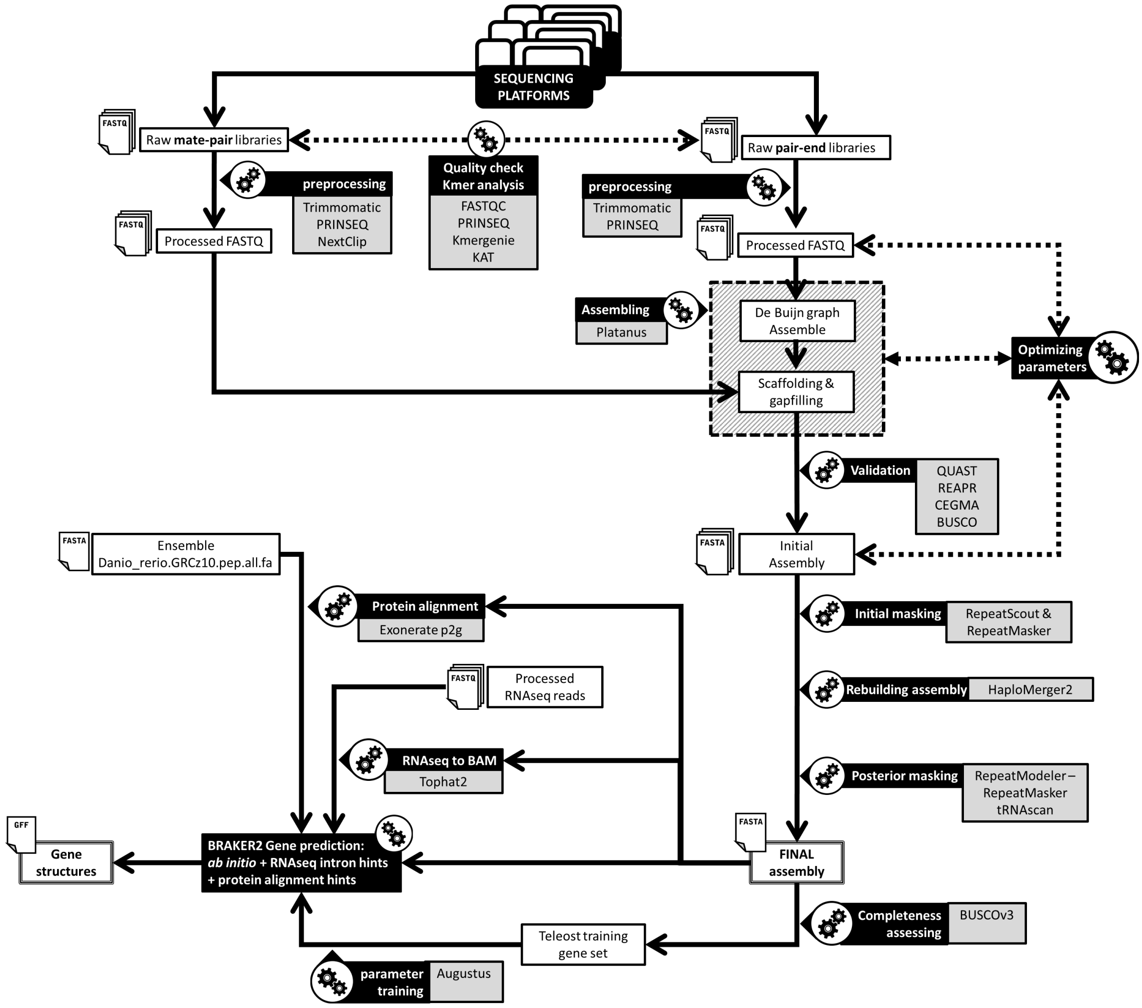

Figure S1. Genome assembly, annotation, and validation pipeline in *Pangasianodon hypophthalmus*. Gray boxes show used software for each analysis. Black boxes indicate processing steps. White boxes show data for input or output.

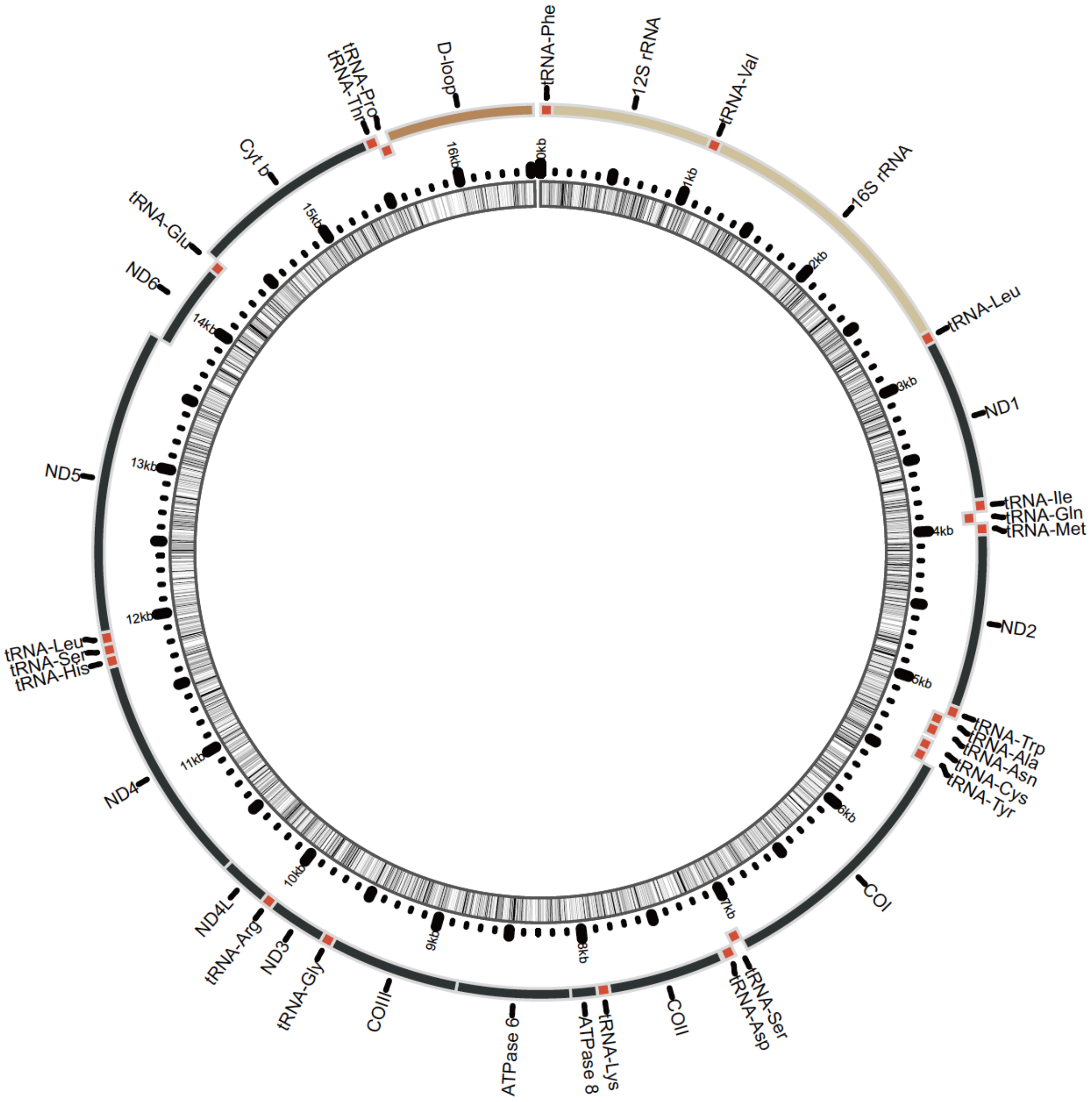

Figure S2. Complete mitochondrial genome of striped catfish, *Pangasianodon hypophthalmus*. Using the Mitofish database [28], 37 genes were predicted in the probable circular genome.
